# Protein nutrition in the ICU: a Delphi exercise to highlight knowledge and opinions of different professional groups involved in patient critical care

**DOI:** 10.1186/s40795-026-01314-3

**Published:** 2026-04-18

**Authors:** Mohamed A Mohamed, Bethan E Phillips, John P Williams

**Affiliations:** 1 Centre of Metabolism, Ageing and Physiology (COMAP), MRC-Versus Arthritis Centre of Musculoskeletal Ageing Research (CMAR) and Nottingham NIHR Biomedical Research Centre, School of Medicine, University of Nottingham, Derby, UK; 2 Department of Anaesthetics and Intensive care, Royal Derby Hospital, Derby, UK

**Keywords:** Protein nutrition, Adjuvant exercise, Critical illness, Patient-centred outcomes, Conventional clinical outcomes

## Abstract

**Background:**

The current literature is lacking in relation to the impact of protein nutrition, with and without adjuvant exercise, on patient centred outcomes including muscle recovery following critical illness. The literature has shown that skeletal muscle health is significantly affected in critical illness with potential negative consequences on physical function and quality of life. This Delphi study aims to establish a consensus opinion for the benefits of protein supplementation alone or as part of multicomponent intervention along with physical exercise in critically ill patients through its iterative process to refine expert opinions with regards of this subject.

**Methodology:**

We conducted this Delphi survey among different groups of experts with extensive experience in critical care management specifically in the area of nutrition and physical rehabilitation and prespecified a threshold of 80% of agreement or disagreement to accept or reject statements respectively; allowing for consensus to be achieved. Anonymous feedback was employed to refine statements across consequent rounds. Statistical stability was also tested to verify the consistency of responses.

**Results:**

The study included 60 statements out of which 18 statements reached agreement (>/= 80%) and only 12 were identified at the near-consensus level (>/= 70%) across the three rounds. It was agreed that physical function, post critical illness quality of life and muscle centred outcomes comprising muscle mass and strength could be enhanced in response to protein provision and adjuvant exercise interventions. However, consensus could not be reached on the most appropriate protein dosage, timing of administration nor on its impact on conventional clinical outcomes.

**Conclusion:**

This study is the first to seek a consensus on the potential benefits of optimal protein provision with and without concomitant exercise on various clinical and patient-centred aspects in the critically ill population. Consensus was attained for a limited number of statements, suggesting that further large robust clinical trials are still required.

**Supplementary Information:**

The online version contains supplementary material available at 10.1186/s40795-026-01314-3.

## Introduction

Skeletal muscle health is negatively impacted by critical illness. Moreover, it is well recognised that both respiratory and limb muscles are susceptible to wasting during acute illness, which in turn can result in significant adverse effects on various conventional and patient-centred outcomes [1].

Acquired critical care illness weakness (ACCIW) may stem from 3 different pathological entities:

i) critical illnesspoly neuropathy (CIP),
ii) critical illness myopathy (CIM) or
iii) a combination of both known as critical illness neuromyopathy [2–4].

Myopathic ACCIW is related to several interacting elements [1]. For instance, disuse atrophy [5]- a major component of anabolic resistance [6], together with unbalanced protein metabolomics [7] are considered the main driving factors of muscle wasting in ICU. These factors themselves are driven by several other components all of which are interacting contributors to myopathy in ICU: exaggerated inflammatory responses [8]; circulatory insufficiency [3, 4]; mitochondrial failure along with disinhibited free radicals [9]; autophagy inadequacy [10]; disorganised intracellular and muscle membrane ionic distribution especially, sodium [11] and calcium [12], and lastly lost coordination between nervous and muscular systems [13].

With muscle atrophy a key aspect of ACCIW, it is important to appreciate that muscle mass maintenance is, in health, controlled by a dynamic equilibrium between muscle protein synthesis (MPS) and muscle protein breakdown (MPB) [14], with protein intake the major anabolic driver [15]. Targeted treatment to protein malnutrition is therefore gaining interest in critical care settings. As such, whilst it is advocated that a minimum protein supply of 0.8 g/kg/day is required to achieve at least zero nitrogen balance [16], current recommendations exhibit significant variability from (1.2–1.5 g/kg/day) [17–19] to (2.0–2.5 g/kg/day) [20]. Strikingly, less than half of the lower recommended protein dose is administered to critically unwell patients in day-to-day clinical practice [21].

Similarly, when to start nutrition, including the protein component, on the ICU is keenly debated amongst ICU researchers. It is also worth pointing out that while, in patients with low risk of malnutrition, ESPEN supports prescription of full enteral nutrition (EN) and parenteral nutrition (PN) within three to seven days, in severe malnutrition whereby EN is contraindicated, early and progressively advanced initiation of PN may be administered as a therapeutic alternative to withholding nutritional support [17]. However, ASPEN and SCCM recommend that, in critically ill patients at low nutritional risk, permissive underfeeding may be tolerated during the first week of ICU admission when EN is not feasible, with deferral of PN [20].

It is also worth highlighting that tailoring protein supplementation according to the nutritional status of the admitted critically ill patients via nutrition assessment tools e.g., NUTRIC (Nutritional Risk in Critically ill) / modified NUTRIC, NRS-2002 (Nutrition Risk Screening-2002) and subjective global assessment (SGA), and monitoring muscle structure and function via quantitative and / or qualitative measures of skeletal muscle e.g., muscle ultrasound (US) [22] and Bioelectrical impedance assessment (BIA) [23] throughout the trajectory of critical illness have gained interest among researchers and clinicians in the critical care settings. It was demonstrated by Mendes et al., that high NUTRIC score has been strongly associated with higher morbidity and mortality risks [24]. Furthermore, NUTRIC score is endorsed by ASPEN for prognostic stratification and decision of initiation of nutrition [20].

Interestingly, muscle US and BIA have progressively evolved over the recent years toward the establishment of well-defined criteria to monitor muscle mass changes in critical care settings. Briefly, Mourtzakis et al., categorised muscle US measures into quantitative parameters including cross sectional area and muscle thickness, and qualitative parameters such as fibre length, pennation angle and fatty infiltration [22]. In parallel, calculated indices particularly, phase angle, as a surrogate marker of cellular health and integrity, using BIA have been recently proved to be valuable predictors of ICU mortality [25, 26]. Further, Looijaard et al., advocated that BIA-based body composition measures might be more informative when interpreted either very early at ICU admission or later following discharge to minimise the confounding effects of major fluid shifts [23].

Likewise, exploring the evidence for contractile activity as an intervention to mitigate ACCIW, findings remain mixed [27–31]. For example, Doiron and colleagues showed that apart from amelioration of delirium in one study, early mobilisation in the ICU does not elicit benefit, with parameters explored in this study including muscle strength, physical performance and all-cause mortality [27]. However, another meta-analysis indicated positive results of physical intervention in the ICU concerning muscle strength, quality of life and physical fitness [28].

More research [30, 32] is emerging on the impact of combining adequate protein nutrition with physical rehabilitation in critically unwell patients. To exemplify, one study, exploring the impact of a combined nutrition and physical activity approach was conducted by Azevedo and colleagues [30], who found that combining resistance exercise with optimal protein intake improved post-discharge physical quality of life and overall mortality. Another study which is still ongoing in this space is the NEXIS trial (Nutrition and Exercise in Critical Illness Trial) which test the influence of combined treatments on the physical performance in the form of assessing 6MWD at hospital discharge and other secondary outcomes including muscle strength.

The overall aim of this Delphi is therefore to establish a consensus among different expert groups on under-researched aspects of protein nutrition and adjunctive physical exercise in critically ill patients. The current literature is lacking in a number of areas related to this aim. Specifically, this Delphi study aims to explore the opinions of different expert groups involved in the care of ICU patients, not only in relation to different protein dosing regimens and the timing of nutritional intervention but also nutrition delivery methods. Furthermore, this Delphi will seek expert insight on the influence of optimum protein nutrition and/or tailored exercise protocols for critically ill patients across an array of clinical and patient-centred outcomes.

## Materials and methods

### Study type

A Delphi study, consisting of 3 consequent rounds, was conducted between November 2024 and March 2025 among 3 different clinical groups involved in providing care to patients in ICU as shown in supplementary files 1,2 and 3. The benefit of this iterative methodology is to affirm stability of participants’ responses which is a sign of consistency across Delphi rounds. While ensuring the stability of responses were maintained, a limitation of 3 rounds was implemented by the research team to mitigate participant attrition and establish a balance between the comprehensiveness of the data gathered and panel members retention rates.

### Panel participants

The target panel of participants comprises senior intensivists, dieticians and physiotherapists with significant expertise in critical illness management including, but not limited to, nutrition and/or physical rehabilitation. In terms of geographical distribution, the panel was recruited from the UK and across the European region. Recruitment was facilitated by invitations sent out on social media sites and to specific professional groups (royal college of intensive care newsletter and special interest groups). Thereafter, responses across the 3 rounds were collected via email invitations using Microsoft Forms.

### Delphi procedure

The first-round survey was constructed of 3 main domains, each of which comprised a number of constructed statements to represent (1) knowledge regarding muscle maintenance in ICU, (2) opinions about protein intake in ICU, and (3) opinions about exercise (adjuvant to protein nutrition) in ICU. Subsequently, the further 2 rounds comprised only the domains of opinions about protein nutrition and adjuvant exercise in ICU.

A 5-point Likert scale (strongly agree, agree, neutral, disagree and strongly disagree plus an option of “unsure”) was employed for scoring. Additionally, space for free text commentary was provided for each domain to enable participants to contextualise their responses.

Following each round, anonymous collated feedback of responses was sent to all participants with the next round statements. For each round, participants were given 4 weeks to complete the survey, with two reminder emails sent during this period before the round was closed. An 80% threshold was set for the acceptance or rejection of statements (i.e. statements scoring at least 80% strongly agree/agree were accepted, and statements scoring at least 80% strongly disagree/disagree were rejected).

To ensure convergence towards consensus, accepted and rejected statements were excluded from subsequent rounds. This iterative refinement facilitated focus on areas where consensus was not reached. Further, in response to experts’ feedback, several statements that lacked consensus were rephrased for enhanced clarity. Additionally, coefficient of variation (CV) was calculated from the mean and the standard deviation of responses to each item to evaluate the answers stability across the 3 rounds of our survey. A cut-off value of 0.2 was applied as the threshold for the absolute difference between CVs of each statement between 2 consequent rounds to verify stability as previously outlined [33, 34].

We conducted a systematic review and meta-analysis [35] prior to this study and showed under-representation of this topic in the literature and that rigorous future research is deemed necessary to draw firm conclusions.

### Ethical considerations

Ethical approval was granted by the University of Nottingham Faculty of Medicine and Health Sciences Research Ethics Committee (FMHS 193–0524). Informed consent after provision of text which explained the nature of the study and exact role of the panel members was obtained from all participants before they completed any activity. All data and responses were anonymised to maintain confidentiality throughout the study duration.

## Results

### Study panel members

A total of 74 participants consented to start this activity comprising 26% senior intensivists, 51% dieticians and 23% physiotherapists. Owing to attrition, the second and third rounds were completed by 54 (31% intensivists, 50% dieticians and 19% physiotherapists) and 41 respondents (34.1% intensivists, 48.8% dieticians and 17.1% physiotherapists), respectively with a final response rate of ~ 55.4%.

### First round results

A total of 60 statements (including 3 knowledge questions and 57 questions with regards opinions of protein nutrition and adjuvant exercise in ICU) were included in the first round, from which 13 statements achieved our pre-specified level of consensus (80%) and as such were not revised or evaluated further in subsequent rounds.

In the domain of opinions about protein intake in ICU, experts agreed that optimal protein nutrition could mitigate muscle mass losses and enhance wound healing in ICU. They also disagreed that optimal protein nutrition could decrease muscle mass and function or worsen post critical illness physical function and quality of life. In the domain of opinions about exercise adjuvant to protein nutrition in ICU, experts were in agreement that it could increase muscle function, mitigate muscle mass loss and improve physical function following ICU discharge. However, aligned with responses in the domain of optimum protein nutrition, they rejected that it could decrease muscle mass and function, and worsen physical function and quality of life after resolution of critical illness. The full results for round one can be seen in Table 1.

**Table 1:** Round 1 - First round results

	Agree (%)	Neutral (%)	Disagree (%)	Unsure (%)	Outcome (%)
Knowledge and confidence regarding muscle maintenance in the ICU					
I am confident prescribing protein nutrition for the ICU patients	64.9%	8.1%	27%	-	-
I am aware of skeletal muscle mass assessment tools in the ICU environment	55.4%	17.6%	26%	-	-
I am aware of skeletal muscle function assessment tools in the ICU environment	52.7%	27%	20.3%	-	-
Opinions about protein intake in the ICU					
Protein dosage in the ICU should be:					
</=0.8 g/kg body weight	9.5%	6.8%	60.8%	23%	Round 2
0.8–1.2 g/kg body weight	43.2%	14.9%	19%	23%	Round 2
1.2–1.5 g/kg body weight	55.4%	4.1%	6.8%	18.9%	Round 2
>/=1.5 g/kg body weight	28.4%	13.5%	27%	31.1%	Round-2
Optimal protein intake could impact patient-centred outcomes in the following ways:					
Increase muscle mass	54%	17.6%	23%	5.4%	Round-2
Decrease muscle mass	6.8%	8.1%	82.5%	2.7%	Rejected
Increase muscle function	66.2%	14.9%	12.2%	6.8%	Round-2
Decrease muscle function	4.1%	9.5%	81.1%	5.4%	Rejected
Mitigate muscle mass losses	90.5%	4.1%	2.7%	2.7%	Accepted
Mitigate muscle function losses	74.4%	9.5%	7.2%	8.1%	Round-2
Improve physical function following discharge	75.7%	12.2%	4.1%	8.1%	Round-2
Worsen physical function following discharge	5.4%	6.8%	83.8%	4.1%	Rejected
Improve quality of life following discharge	68.9%	16.2%	5.4%	9.5%	Round-2
Worsen quality of life following discharge	2.7%	9.5%	81.1%	6.8%	Rejected
Reduce risk of discharge to rehabilitation facilities	37.8%	36.5%	10.9%	14.9%	Round-2
Increase risk of discharge to rehabilitation facilities	5.5%	23%	62.1%	9.5%	Round-2
Optimal protein intake could impact conventional clinical outcomes in the following ways:					
Reduce ICU mortality	62.2%	21.6%	6.8%	9.5%	Round-2
Increase ICU mortality	5.5%	13.5%	73%	8.1%	Round-2
Shorten ICU length of stay	59.5%	24.3%	5.5%	10.8%	Round-2
Lengthen ICU length of stay	2.7%	21.6%	66.2%	9.5%	Round-2
Increase ventilator free days	56.7%	23%	9.5%	10.8%	Round-2
Decrease ventilator free days	8.9%	16.2%	60.8%	14.1%	Round-2
Decrease risk of sepsis	35.2%	37.8%	2.7%	24.3%	Round-2
Increase risk of sepsis	0%	24.3%	56.7%	18.9%	Round-2
Negatively impact renal function	12.2%	27%	40.6%	20.3%	Round-2
Positively impact renal function	18.9%	39.2%	16.2%	25.7%	Round-2
Enhance wound healing	83.8%	6.8%	1.4%	8.1%	Accepted
Worsen wound healing	0%	10.8%	77%	12.2%	Round-2
Following ICU admission protein nutrition should start:					
Within < 24 h	35.1%	24.3%	25.7%	14.9%	Round-2
Within 24–48 h	62.2%	13.5%	10.9%	13.5%	Round-2
Within 48–72 h	37.9%	16.2%	28.4%	17.6%	Round-2
> 72 h	20.3%	14.9%	48.7%	16.2%	Round-2
Early protein supplementation in the ICU could:					
Be harmful for septic patients	23%	21.6%	27%	28.4%	Round-2
Be associated will less nutritional deficits	58.1%	13.5%	8.1%	20.3%	Round-2
Improve patient-centred functional outcomes	67.6%	10.8%	6.8%	14.9%	Round-2
Improve conventional clinical outcomes	58.1%	17.6%	8.1%	16.2%	Round-2
Considering intermittent versus continuous protein provision in the ICU.					
Intermittent, compared to continuous, provision could enhance the cellular process of muscle building	25.7%	32.4%	5.4%	36.5%	Round-2
Continuous, compared to intermittent, provision is more feasible in the ICU environment	44.6%	16.2%	12.2%	27%	Round-2
Intermittent, compared to continuous, enteral provision could have a negative effect on gastric function *(e.g.*,* vomiting and aspirates)*	16.2%	33.8	17.6%	32.4%	Round-2
‘Exercise’ (contractile activity) adjuvant to optimal protein intake could impact patient-centred functional outcomes in the following ways:					
Increase muscle mass	75.7%	13.5%	6.8%	4.1%	Round-2
Decrease muscle mass	1.4%	8.1%	83.8%	6.8%	Rejected
Increase muscle function	85.1%	8.1%	2.8%	4.1%	Accepted
Decrease muscle function	2.7	9.5%	83.8%	4.1%	Rejected
Mitigate muscle mass losses	87.8%	6.8%	1.4%	4.1%	Accepted
Mitigate muscle function losses	70.3%	9.5%	16.3%	4.1%	Round-2
Improve physical function following discharge	82.4%	6.8%	2.7%	8.1%	Accepted
Worsen physical function following discharge	2.7%	9.5%	82.4%	5.4%	Rejected
Improve quality of life following discharge	73%	14.9%	1.4%	10.8%	Round-2
Worsen quality of life following discharge	1.4%	10.8%	82.4%	5.4%	Rejected
Reduce risk of discharge to rehabilitation facilities	54%	24.3%	6.8%	14.9%	Round-2
Increase risk of discharge to rehabilitation facilities	4.1%	23%	63.5%	9.5%	Round-2
‘Exercise’ (contractile activity) adjuvant to optimal protein intake could impact conventional clinical outcomes in the following ways:					
Reduce ICU mortality	43.3%	23%	6.8%	27%	Round-2
Increase ICU mortality	4.1%	20.3%	55.4%	20.3%	Round-2
Shorten ICU length of stay	56.7%	17.6%	2.8%	23%	Round-2
Increase ICU length of stay	1.4%	14.9%	66.2%	17.6%	Round-2
Increase ventilator free days	51.3%	17.6%	8.1%	23%	Round-2
Decrease ventilator free days	6.8%	17.6%	55.4%	20.3%	Round-2

### Second round results

Forty-four statements were carried forward to the second round after exclusion of the statements which reached our predetermined threshold of consensus, and the statements under the domain of knowledge regarding muscle maintenance in ICU which were only ever intended as single-round items. In the domain of opinions about protein intake in ICU, 5 statements which were near consensus (70–80%) in round one achieved a robust consensus in round two. Experts agreed that adequate protein nutrition in critical illness could mitigate muscle function loss and improve post-discharge physical function. They also agreed that concomitant contractile activity could improve quality of life and mitigate muscle function loss. They rejected that adequate protein intake could worsen wound healing in ICU patients. The full results for round two can be seen in Table 2.

**Table 2:** Round 2 - Second round results

	Agree (%)	Neutral (%)	Disagree (%)	Unsure (%)	Outcome (%)
I. Opinions about protein intake in the ICU					
Protein consumption in the ICU should be:					
</=0.8 g/kg body weight	19.2%	3.8%	71.2%	5.8%	Round-3
0.8–1.2 g/kg body weight	44.2	23.1%	11.5%	21.2%	Round-3
1.2–1.5 g/kg body weight	61.5%	7.7%	7.7%	23.1%	Round-3
>/=1.5 g/kg body weight	9.6%	17.3%	36.5%	36.5%	Round-3
Adequate protein provision could affect patient-focused outcomes regarding:					
Increase muscle mass	44.2%	30.8%	19.2%	5.8%	Round-3
Increase muscle function	48.1%	26.9%	17.3%	7.7%	Round-3
Mitigate muscle function loss	88.4%	7.6%	1.9%	1.9%	Accepted
Improve quality of life following discharge	73.1%	15.4%	3.8%	7.7%	Round-3
Improve physical function following discharge	82.6%	7.6%	3.8%	5.7%	Accepted
Reduce risk of discharge to rehabilitation facilities	51.9%	26.9%	7.7%	13.5%	Round-3
Increase risk of discharge to rehabilitation facilities	17.3%	11.5%	65.4%	5.8%	Round-3
Adequate protein provision could affect standard clinical outcomes regarding:					
Reduce ICU mortality	48.1%	23.1%	13.5%	15.4%	Round-3
Increase ICU mortality	1.9%	21.2%	59.6%	17.3%	Round-3
Shorten ICU length of stay	42.3%	34.6%	9.6%	13.5%	Round-3
Lengthen ICU length of stay	3.8%	13.5%	67.3%	15.4%	Round-3
Increase ventilator free days	44.2%	19.2%	19.2%	17.3%	Round-3
Decrease ventilator free days	5.8%	19.2%	59.6%	15.4%	Round-3
Decrease risk of sepsis	25%	32.7%	19.2%	23.1%	Round-3
Increase risk of sepsis	63.5%	9.6%	1.9%	25%	Round-3
Negatively impact renal function	7.7%	21.2%	51.9%	19.2%	Round-3
Positively impact renal function	19.2%	34.6%	30.8%	15.4%	Round-3
Worsen wound healing	0%	3.8%	92.3%	3.8%	Rejected
Initiation of protein provision post ICU discharge could be:					
Within < 24 h	21.2%	38.5%	26.9%	13.5%	Round-3
Within 24–48 h	63.5%	11.5%	11.5%	13.5%	Round-3
Within 48–72 h	34.6%	21.2%	32.7%	11.5%	Round-3
> 72 h	21.2%	5.8%	61.5%	11.5%	Round-3
Early amino acids supplementation in the ICU could:					
Adversely affect septic patients	28.8%	28.8%	21.2%	21.2%	Round-3
Be correlated with reduced nutritional deficiencies	61.5%	15.4%	7.7%	15.4%	Round-3
Improve patient-focused functional outcomes	48.1%	25%	11.5%	15,4%	Round-3
Improve standard clinical outcomes	38.5%	25%	13.5%	23.1%	Round-3
Considering boluses of protein delivery versus steady state delivery in the ICU.					
boluses, compared to steady state delivery, could enhance the cellular process of muscle building	30.8%	21.2%	7.7%	40.4%	Round-3
Steady state delivery, compared to boluses, is more feasible in the ICU environment	57.7%	17.3%	11.5%	13.5%	Round-3
boluses, compared to steady state enteral delivery could have a negative effect on gastric function *(e.g.*,* vomiting and aspirates)*	23.1%	32.7%	15.4%	28.8%	Round-3
‘Exercise’ (contractile activity) concomitant to adequate protein intake could affect patient-focused outcomes regarding:					
Increase muscle mass	55.8%	21.2%	15.4%	7.7%	Round-3
Mitigate muscle function loss	86.5%	9.6%	5.7%	3.8%	Accepted
Improve quality of life following discharge	80.7%	9.6%	3.8%	5.7%	Accepted
Reduce risk of discharge to rehabilitation facilities	75%	11.5%	5.8%	7.7%	Round-3
Increase risk of discharge to rehabilitation facilities	11.5%	21.2%	61.5%	5.8%	Round-3
‘Exercise’ (contractile activity) concomitant to adequate protein intake could affect standard clinical outcomes regarding:					
Reduce ICU mortality	46.2%	26.9%	7.7%	19.2%	Round-3
Increase ICU mortality	0%	19.2%	65.4%	15.4%	Round-3
Shorten ICU length of stay	50%	25%	3.8%	21.2%	Round-3
Increase ICU length of stay	0%	17.3%	69.2%	13.5%	Round-3
Increase ventilator free days	44.2%	21.2%	17.3%	17.3%	Round-3
Decrease ventilator free days	7.7%	17.3%	55.8%	19.2%	Round-3

### Third round results

Thirty-nine statements were advanced to the third round. Although no statement reached consensus, an additional 7 statements were identified as near consensus (70–80%) at the end of round three. The full results for round three can be seen in Table 3.

**Table 3:** Round 3 - Third round results

	Agree (%)	Neutral (%)	Disagree (%)	Unsure (%)	Outcome (%)
I. Opinions about protein intake in the ICU					
*Target* protein dosage in the ICU should be:					
</=0.8 g/kg body weight	4.8%	19.5%	63.4%	12.2%	No consensus reached
0.8–1.2 g/kg body weight	41.4%	29.3%	16.3%	12.2%	No consensus reached
1.2–1.5 g/kg body weight	70.7%	7.3%	7.3%	14.6%	No consensus reached
>/=1.5 g/kg body weight	17.1%	24.4%	41.5%	17.1%	No consensus reached
Adequate protein provision could affect patient-focused outcomes regarding:					
Build up muscle mass	48.8%	29.3%	19.5%	2.4%	No consensus reached
Improve muscle function	60.9%	19.5%	14.7%	4.9%	No consensus reached
Enhance quality of life following discharge	73.1%	17.1%	4.8%	4.9%	No consensus reached
Decrease risk of discharge to rehabilitation facilities	73.2%	14.6%	7.3%	4.9%	No consensus reached
Elevate risk of discharge to rehabilitation facilities	19.5%	9.8%	65.9%	4.9%	No consensus reached
Adequate protein provision could affect standard clinical outcomes regarding:					
Improve ICU mortality	48.8%	29.3%	12.2%	9.8%	No consensus reached
Worsen ICU mortality	7.3%	9.8%	75.6%	7.3%	No consensus reached
Decrease ICU length of stay	56.1%	26.8%	7.3%	9.8%	No consensus reached
Increase ICU length of stay	12.2%	9.8%	73.2%	4.9%	No consensus reached
Increase ventilation dependency	7.3%	9.8%	78%	4.9%	No consensus reached
Decrease ventilation dependency	53.7%	24.4%	12.2%	9.8%	No consensus reached
Reduce risk of sepsis	26.8%	43.9%	14.6%	14.6%	No consensus reached
Increase risk of sepsis	0%	31.7%	61%	7.3%	No consensus reached
Worsen renal function	19.5%	31.7%	39.1%	9.8%	No consensus reached
Improve renal function	19.5%	43.9%	24.4%	12.2%	No consensus reached
Following ICU admission *target* protein nutrition should be reached:					
Within < 24 h	9.7%	7.3%	78%	4.9%	No consensus reached
Within 24–48 h	24.4%	14.6%	53.6%	7.3%	No consensus reached
Within 48–72 h	43.9%	22%	26.8%	7.3%	No consensus reached
> 72 h	61%	14.6%	17.1%	7.3%	No consensus reached
Early amino acids supplementation in the ICU could:					
Negatively affect septic patients	29.2%	31.7%	26.8%	12.2%	No consensus reached
Offer more nutritional sufficiency	46.3%	29.3%	12.2%	12.2%	No consensus reached
Enhance patient-centred functional outcomes	43.9%	26.8%	12.2%	17.1%	No consensus reached
Enhance conventional clinical outcomes	39%	24.4%	17%	19.5%	No consensus reached
Considering boluses of protein delivery versus steady state delivery in the ICU.					
boluses, compared to steady state delivery, provision could improve muscle synthesis	22%	39%	9.8%	29.2%	No consensus reached
Steady state delivery, compared to boluses, provision is more practicable in the ICU settings	53.6%	22%	7.3%	17.1%	No consensus reached
boluses, compared to steady state, enteral provision could worsen gastric function (e.g., vomiting and higher risk of aspiration)	34.1%	29.3%	12.2%	24.4%	No consensus reached
‘Exercise’ (contractile activity) concomitant to adequate protein intake could affect patient-focused outcomes regarding:					
Build up muscle mass	56.1%	22%	19.5%	2.4%	No consensus reached
Decrease risk of discharge to rehabilitation facilities	68.3%	24.4%	2.4%	4.9%	No consensus reached
Worsen risk of discharge to rehabilitation facilities	7.3%	14.6%	73.2%	4.9%	No consensus reached
‘Exercise’ (contractile activity) concomitant to adequate protein intake could affect standard clinical outcomes regarding:					
Decrease ICU mortality	46.4%	29.3%	14.6%	9.8%	No consensus reached
Elevate ICU mortality	7.3%	9.8%	75.6%	7.3%	No consensus reached
Decrease ICU length of stay	70.8%	19.5%	7.3%	2.4%	No consensus reached
Lengthen ICU length of stay	2.4%	14.6%	78%	4.9%	No consensus reached
Decrease ventilation dependency	63.4%	24.4%	4.9%	7.3%	No consensus reached
Increase ventilation dependency	0%	19.5%	75.6%	4.9%	No consensus reached

### Delphi final results

At conclusion of the survey, consensus was achieved for 18 statements (Table 4) with a further 12 statements identified as being near consensus (Table 5).

**Table 4:** Consensus statements - Statements reached consensus of 80%

Statement	Expert opinion	Round	Level of consensus
Optimal protein intake / Adequate protein provision could			
Decrease muscle mass	Disagree	First round	82.5%
Decrease muscle function	Disagree	First round	81.1%
Mitigate muscle mass losses	Agree	First round	90.5%
Worsen physical function following discharge	Disagree	First round	83.8%
Worsen quality of life following discharge	Disagree	First round	81.1%
Enhance wound healing	Agree	First round	83.8%
Mitigate muscle function loss	Agree	Second round	88.4%
Improve physical function following discharge	Agree	Second round	82.6%
Worsen wound healing	Disagree	Second round	92.3%
‘Exercise’ (contractile activity) adjuvant (concomitant) to optimal (adequate) protein intake could			
Decrease muscle mass	Disagree	First round	83.8%
Increase muscle function	Agree	First round	85.1%
Decrease muscle function	Disagree	First round	83.8%
Mitigate muscle mass losses	Agree	First round	87.8%
Improve physical function following discharge	Agree	First round	82.4%
Worsen physical function following discharge	Disagree	First round	82.4%
Worsen quality of life following discharge	Disagree	First round	82.4%
Mitigate muscle function loss	Agree	Second round	86.5%
Improve quality of life following discharge	Agree	Second round	80.7%

**Table 5:** Near consensus statements - Statements reached near-consensus of 70%

Statement	Expert opinion	Level of consensus
Target protein dosage in the ICU should be 1.2–1.5 g/kg body weight	Agree	70.7%
Following ICU admission target protein nutrition should be reached within 24 h	Disagree	78%
Optimal (Adequate) protein intake (provision) could worsen ICU mortality	Disagree	75.6%
Optimal (adequate) protein intake (provision) could increase ICU length of stay	Disagree	73.2%
Optimal (adequate) protein intake (provision) could increase ventilation dependency	Disagree	78%
Optimal (adequate) protein intake (provision) could enhance quality of life following discharge	Agree	73.1%
Optimal (adequate) protein intake (provision) could decrease risk of discharge to rehabilitation facilities	Agree	73.2%
Exercise’ (contractile activity) adjuvant (concomitant) to optimal (adequate) protein intake (provision) could worsen risk of discharge to rehabilitation facilities	Disagree	73.2%
Exercise’ (contractile activity) adjuvant (concomitant) to optimal (adequate) protein intake (provision) could decrease ICU length of stay	Agree	70.3%
Exercise’ (contractile activity) adjuvant (concomitant) to optimal (adequate) protein intake (provision) could elevate ICU mortality	Disagree	75.6%
Exercise’ (contractile activity) adjuvant (concomitant) to optimal (adequate) protein intake (provision) could lengthen ICU length of stay	Disagree	78%
Exercise’ (contractile activity) adjuvant (concomitant) to optimal (adequate) protein intake (provision) could increase ventilation dependency	Disagree	75.6%

### Delphi responses stability

The coefficient of variation (CV) was computed from the responses to each item to assess the stability of responses. The absolute differences in CV between the vast majority of projections from successive rounds were consistently found to be less than 0.2 as shown in supplementary file 4, indicating a high level of response stability across the three rounds [33, 34].

## Discussion

This Delphi study was conducted to explore clinical opinions on skeletal muscle health during critical illness since this area is underrepresented by the existing research. A set of consensus statements by experts from different clinical backgrounds has been generated, with potential to inform future studies into protein nutrition and adjuvant exercise interventions in critical care settings. To the best of our knowledge, such a consensus has not been presented previously in the literature.

Following 3 rounds of expert responses, optimised protein intake either alone or with adjunctive contractile activity was agreed to enhance some structural and functional aspects of skeletal muscle throughout the trajectory of critical illness and improve quality of life and less reliance on long-term clinical recovery services after critical illness. Briefly, both exclusive adequate protein provision and with concomitant physical rehabilitation were agreed for their potential to mitigate muscle mass and function losses, which are well-known significant sequelae of critical illness [1]. Supporting these findings, Ferrie et al., demonstrated increased muscle volume in a high protein group of ICU patients (1.2 g/kg/day) when compared to provision of lower protein doses (0.8 g/kg/day) [36]. Likewise, a meta-analysis conducted by Tipping and colleagues [31] demonstrated a positive correlation between active rehabilitation and muscle strength in critically unwell patients, a finding which has been further substantiated by few randomised controlled trials [37–39].

While out of the scope of this study, quality of supplied proteins has started to draw researchers’ attractions as determinants of critical illness outcomes [40], particularly, leucin and its active metabolite Beta-Hydroxy Beta-Methylbutyrate (HMB) which have been shown to have key roles in muscle protein anabolism in several conditions of anabolic resistance [40]. In a recent review, essential amino acids and branched chain amino acids have been found to stimulate muscle protein synthesis, yet without muscle strength positive impact and marginal benefits of HMB provision in the critically ill population [41].

In relation to the post-ICU outcomes of quality of life and physical function, whilst no agreement was obtained on the impact of optimum protein supply alone on the quality of life in the critically ill population, a consensus from the panel members was reached that concomitant protein and exercise interventions could enhance both quality of life and physical function after discharge from ICU, a perspective supported by findings from various trials. For example, Azevedo and colleagues [30] conducted a study looking at the influence of high protein intake with resistance exercise on various conventional and patient centred clinical outcomes and reported enhanced survival rate and physical quality of life. Nevertheless, the PRECISe trial [42] demonstrated worse quality-of-life outcomes with high protein provision; notably, no physical exercise component formed part of the intervention.

Despite the overarching theme appearing from the statements that did reach consensus supporting muscle centric outcomes e.g., mitigation of muscle function and mass losses for adequate protein supply with and without adjuvant contractile activity, consensus about the impact of protein +/- adjuvant exercise was not achieved for the vast majority of statements in terms of conventional clinical outcomes on the ICU (i.e., mortality, length of stay, ventilator-free days, sepsis risk, renal function). Indeed, the only consensus achieved was on the positive impact of optimal protein nutrition on wound healing. This lack of consensus does appear to reflect the lack of consistent findings reported in the literature. For example, some studies showed improved survival rates linked to adequate protein supplementation [43, 44] including with adjuvant exercise [30], whilst others do not [44]. To elucidate further, it was found that lower risk of mortality was observed in the high protein cohort if exclusively non-septic and protected from energy overfeeding, however no significant benefit was observed in energy over-fed and septic patients [44]. However, findings from the recent EFFORT and TARGET protein trials [45, 46] did not demonstrate significant improvement in conventional outcomes including mortality in association with augmented protein supplementation. Moreover, in the context of acute lung injury, the EDEN trial reported no significant differences between trophic and full nutrient supply in terms of mortality and mechanical ventilation dependency [47].

Interestingly, our findings appear to show a lack of awareness of the connection between muscle-centric and patient-centred/clinical outcomes, which was previously demonstrated in the literature whereby high protein provision led to favourable fatigue scores along with positive structural and functional muscle outcomes [36] Furthermore, Matsushima et al., surmised that accelerated physical recovery following ICU discharge in the high protein group was likely attributed to preserved muscle strength [48].

Consistent with the current available evidence regarding protein nutrition in ICU patients suffering from renal impairment, a noticeable divergence of opinions was detected among the experts and no consensus achieved on whether protein nutrition would positively or negatively influence renal function. Although higher protein supply in AKI is still endorsed by international guidelines [49] and has been shown to elicit positive effects in a number of research studies [50, 51], concerns about potential harm remain [46, 52, 53].

Similarly, with respect to timing and dosing of protein nutrition in ICU patients with/at risk of sepsis, conflicting perspectives are represented in the literature, and this was echoed by our lack of consensus for statements in this area. De Waele et al., recommends that high protein provision should be avoided in early stages of septic shock, suggesting a ‘start low and go slow’ approach, with the aim of targeting optimal doses after resolution of the shock state [54]. Conversely, Elke and colleagues found that early initiation of macronutrients, including protein, close to optimal doses are associated with favourable clinical outcomes including mortality and ventilator free days [55].

Further, as endorsed by ESPEN [17], hypocaloric intake (~ 70% of energy expenditure) should be considered in the acute phase of critical illness to avoid overfeeding and potential drawbacks such as higher infection rates, increased length of stay and ventilator dependency [56]. Therefore, recommendations from ESPEN [17] and ASPEN [20] support the use of calorimetry to accurately measure energy expenditure and tailor caloric intake accordingly. Indeed, comprehensive metabolic monitoring is indispensable in malnourished critically ill patients receiving medical nutrition therapy, particularly parenteral nutrition (PN), due to the heightened risk of complications such as refeeding syndrome and hepatic dysfunction. Accordingly, thorough nutritional assessment, accompanied by regular laboratory evaluation of serum electrolytes, glucose levels, and liver function parameters, is strongly recommended [17].

Referring to optimal protein dosage in the critically ill population, only a near consensus level of agreement was achieved. Although roughly 70% of participants selected provision of 1.2–1.5 g/kg/day, only 63% of them disagreed on providing less than 0.8 g/kg/day, seemingly reflective of concerns about protein dosing in specific groups of patients (i.e., renal failure). Indeed, free-text comments provided by our expert panel support this supposition with comments including “*protein doses should be tailored to individual critical care cases*”, *“… based on various cofounders e.g.*,* renal impairment*,* stage of critical illness*,* septic shock and nature of the culprit pathology*”. Further, 1.2–1.5 g/kg/day is the target dosage most potently promoted in the current literature, including the most recent ESPEN guidance whereby this dosage is outlined as optimal for critical care settings [17–19]. Despite this, significant debate does still exist, with emerging recommendations advocating higher doses of 2.0–2.5 g/kg/day which are the minimum doses to provide in highly metabolically active scenarios e.g., severe burn, multiple traumatic cases, undernourished obese patients to meet the heightened metabolic demand pertinent to these situations [57].

As was seen for optimal target protein doses, consensus could not be reached for timing of initiation of protein feeding either, with a majority of 61% of experts agreeing that target protein nutrition should be reached beyond 72 h of ICU admission while 78% of them disagreeing to reach the target within 24 h. This lack of consensus may reflect the lack of agreement between international guidance, and the sparsity of high-quality research studies exploring this paradigm. To exemplify, ASPEN promotes that trophic nutrition could be tolerated for up to 1 week, whilst ESPEN advocates prescription of full-feed within three to seven days of admission to avoid overfeeding in the critically ill population [17, 49].

Overall, our results show that experts have diverging opinions regarding the optimal dose and initiation strategy of protein nutrition and also appear to be unable to reach consensus on benefits on various patient centred and conventional clinical outcomes. Despite this, there is agreement across expert groups involved in the care of ICU groups that optimal protein nutrition may mitigate muscle losses, and improve physical recovery and wound healing, however, agreement on the potential benefit for early or late initiation of protein nutrition is not as apparent.

### Strengths and limitations

There are several strengths to this current study, including the sustained consistency of expert responses across 3 rounds of the survey and the diverse composition of the panel comprising three groups of experts (senior intensivists, dieticians and physiotherapists), each of whom possesses extensive expertise in the field of critical care. However, we conducted three questions in the beginning of the questionnaire to confirm Knowledge and confidence regarding muscle maintenance in the ICU. Furthermore, the results of this study may help direct the framework of future studies, particularly those aim at exploring the impact of protein nutrition and adjunctive physical activity on functional recovery. Despite our attempts to fulfil Delphi methodology requirements, we acknowledge the limitation of participants dropout rate of approximately 45% across the three study rounds. Although participants were given the opportunity to contextualise their responses through free-text comments for each domain, the withdrawal of participants with dissenting views remains a possible contributing factor to attrition in survey studies. However, this level of attrition is fairly common in Delphi studies [58], and herein did not alter the balance of the respondents from the three professional groups compared to those initially recruited. Notwithstanding the reasonably balanced recruitment in a study where enrolment was challenging, the possibility of selection bias arising from the recruitment process cannot be excluded.

## Conclusion

This study is the first to seek a consensus amongst a group of critical care experts on the potential benefits of optimal protein provision with and without concomitant exercise on various clinical and patient-centred aspects in the critically ill population. Consensus was attained for a limited number of statements, particularly those concerning patient centred outcomes. Briefly, whilst optimum protein nutrition with or without adjuvant exercise were agreed by the expert panel to mitigate select muscle centred outcomes e.g., mass and function losses and, post-discharge physical function, there was a consensus that only concomitant interventions might enhance post-discharge quality of life. Large robust clinical trials are still required to support these findings and introduce timely appropriate doses of protein nutrition and whether these interventions could improve other conventional outcomes. It would also be valuable to consider trials incorporating patient reported outcomes measures and long-term follow-up which may provide additional support for our findings. Knowledge gained from such trials would hopefully enhance confidence and consistency in prescribing protein nutrition and physical activity to ICU patients.

## Supplementary Information

Supplementary Material 1.

Supplementary Material 2.

Supplementary Material 3.

Supplementary Material 4.

Supplementary Material 5.

## Data Availability

The datasets analysed during the current study are available from the corresponding author on reasonable request.
