# Supplementary material for: Protein nutrition in the ICU: a Delphi exercise to highlight knowledge and opinions of different professional groups involved in patient critical care
Source: BMC Nutr. 2026 Apr 18;12:101. doi: 10.1186/s40795-026-01314-3 (PMC13220506; doi:10.1186/s40795-026-01314-3)
Supplement: Supplementary file 1 — Supplementary Material 1. [file 40795_2026_1314_MOESM1_ESM.pdf]

Protein nutrition and adjuvant exercise in the ICU

A Delphi exercise to highlight opinions of different professional groups involved in patient critical care

* Required

1. **My email address is** *

2. **Which professional group do you belong to?** *

Intensivist	ICU Dietician	ICU physiotherapist
○	○	○

Opinions about protein intake in the ICU

3. **<u>Target</u> protein dosage in the ICU should be:** *

	Strongly agree	Agree	Neutral	Disagree	Strongly disagree	Unsure
</=0.8 g/kg body weight	○	○	○	○	○	○
0.8-1.2 g/kg body weight	○	○	○	○	○	○
1.2 -1.5 g/kg body weight	○	○	○	○	○	○
>/=1.5 g/kg body weight	○	○	○	○	○	○

4. **Please add any comments to support your answer to the question above.**

5. **Adequate protein provision could affect patient-focused outcomes regarding:** *

	Strongly agree	Agree	Neutral	Disagree	Strongly disagree	Unsure
Build up muscle mass	◯	◯	◯	◯	◯	◯
Improve muscle function	◯	◯	◯	◯	◯	◯
Enhance quality of life following discharge	◯	◯	◯	◯	◯	◯
Decrease risk of discharge to rehabilitation facilities	◯	◯	◯	◯	◯	◯
Elevate risk of discharge to rehabilitation facilities	◯	◯	◯	◯	◯	◯

6. **Please add any comments to support your answer to the question above.**

7. **Adequate protein provision could affect standard clinical outcomes regarding:** *

	Strongly agree	Agree	Neutral	Disagree	Strongly disagree	Unsure
Improve ICU mortality	◯	◯	◯	◯	◯	◯
worsen ICU mortality	◯	◯	◯	◯	◯	◯
Decrease ICU length of stay	◯	◯	◯	◯	◯	◯
Increase ICU length of stay	◯	◯	◯	◯	◯	◯
Increase ventilation dependency	◯	◯	◯	◯	◯	◯
Decrease ventilation dependency	◯	◯	◯	◯	◯	◯
Reduce risk of sepsis	◯	◯	◯	◯	◯	◯
Increase risk of sepsis	◯	◯	◯	◯	◯	◯

Worsen renal function	◯	◯	◯	◯	◯	◯
Improve renal function	◯	◯	◯	◯	◯	◯

8. **Please add any any comments to support your answer to the question above.**

9. **Following ICU admission *target* protein nutrition should be reached:** *

	Strongly agree	Agree	Neutral	Disagree	Strongly disagree	Unsure
Within <24 hours	◯	◯	◯	◯	◯	◯
Within 24-48 hours	◯	◯	◯	◯	◯	◯
Within 48-72 hours	◯	◯	◯	◯	◯	◯
>72 hours	◯	◯	◯	◯	◯	◯

10. **Please add any comments to support your answer to the question above.**

11. **Early amino acids supplementation in the ICU could:** *

	Strongly agree	Agree	Neutral	Disagree	Strongly disagree	Unsure
Negatively affect septic patients	○	○	○	○	○	○
Offer more nutritional sufficiency	○	○	○	○	○	○
Enhance patient-centred functional outcomes	○	○	○	○	○	○
Enhance conventional clinical outcomes	○	○	○	○	○	○

12. **Please add any comments to support your answer to the question above.**

13. **Considering boluses of protein delivery versus steady state delivery in the ICU.** *

	Strongly agree	Agree	Neutral	Disagree	Strongly disagree	Unsure
boluses, compared to steady state delivery, provision could improve muscle synthesis	○	○	○	○	○	○
Steady state delivery, compared to boluses, provision is more practicable in the ICU settings	○	○	○	○	○	○
boluses, compared to steady state, enteral provision could worsen gastric function (e.g., vomiting and higher risk of aspiration)	○	○	○	○	○	○

14. **Please add any comments to support your answer to the question above.**

Opinions about adjuvant exercise in the ICU

15. **'Exercise' (contractile activity) concomitant to adequate protein intake could affect patient-focused outcomes regarding:** *

	Strongly agree	Agree	Neutral	Disagree	Strongly disagree	Unsure
Build up muscle mass	○	○	○	○	○	○
Decrease risk of discharge to rehabilitation facilities	○	○	○	○	○	○
Worsen risk of discharge to rehabilitation facilities	○	○	○	○	○	○

16. **Please add any comments to support your answer to the question above.**

17. **‘Exercise’ (contractile activity) concomitant to adequate protein intake could affect standard clinical outcomes regarding:** *

	Strongly agree	Agree	Neutral	Disagree	Strongly disagree	Unsure
Decrease ICU mortality	◯	◯	◯	◯	◯	◯
Elevate ICU mortality	◯	◯	◯	◯	◯	◯
Decrease ICU length of stay	◯	◯	◯	◯	◯	◯
Lengthen ICU length of stay	◯	◯	◯	◯	◯	◯
Decrease ventilation dependency	◯	◯	◯	◯	◯	◯
Increase ventilation dependency	◯	◯	◯	◯	◯	◯

18. **Please add any comments to support your answer to the question above.**

This content is neither created nor endorsed by Microsoft. The data you submit will be sent to the form owner.

Microsoft Forms
