# Supplementary material for: Protein nutrition in the ICU: a Delphi exercise to highlight knowledge and opinions of different professional groups involved in patient critical care
Source: BMC Nutr. 2026 Apr 18;12:101. doi: 10.1186/s40795-026-01314-3 (PMC13220506; doi:10.1186/s40795-026-01314-3)
Supplement: Supplementary file 2 — Supplementary Material 2. [file 40795_2026_1314_MOESM2_ESM.pdf]

Protein nutrition and adjuvant exercise in the ICU

A Delphi exercise to highlight knowledge and opinions of different professional groups involved in patient critical care

* Required

Consent and personal information

1. **CONSENT STATEMENT**

Research group: Mohamed A Mohamed, Bethan E Phillips and John P Williams

MAM, BEP and JPW are all members of the Centre of Metabolism, Ageing and Physiology (COMAP) at the University of Nottingham, School of Medicine, Derby, UK. MAM and JPW also work for the Department of Anaesthesia and Intensive care at the Royal Derby Hospital, Derby, UK.

Dear Participant,

Many thanks for considering participation in our Delphi study. Before proceeding, we want to ensure that you fully understand the nature of the study and what participation entails. Please read the following information carefully:
Study Description: You are invited to take part in a Delphi study conducted by the above-mentioned research team. The purpose of this study is to gather expert opinions and insight on the impact of protein supplementation and adjuvant exercise on different patient centred and conventional outcomes for patients admitted to an intensive care unit (ICU).

Your Role: As a participant, you will be asked to provide your expert opinion on a series of statements related to the study topic. These should be answered based on your current knowledge and opinion, without further research or consultation with others. A Delphi study involves the revision of questions that do not meet consensus to try and improve agreement. As such, participation in this study will involve completing a maximum of 3 surveys, with subsequent surveys shorter than the one before. It is anticipated that you will be invited via email to complete the second survey approximately 6 weeks after this one. Each survey will be open for 4 weeks.
Confidentiality: Your personal details (email) will only be accessible to the direct research team, and only used for the purpose of inviting you to complete the subsequent surveys that form part of this Delphi study. Your responses (the research data) will be anonymised data via a participant code. Any information shared will be anonymized this way, ensuring that your identity remains protected.
Voluntary Participation: Participation in this study is entirely voluntary, and you may withdraw at any time without providing a reason.
Informed Consent: By agreeing to participate, you acknowledge that you have read and understood the information provided in this consent statement. You are aware of the study's purpose, your role as a participant, and the confidentiality measures in place. If you have any questions or concerns, please do not hesitate to contact the research team at: Mohamed.Mohamed4@nottingham.ac.uk .
Consent: If you agree to participate in this study, please indicate your consent by ticking the button below and proceeding with the survey. Your consent indicates that you are willing to contribute your expertise to this research endeavour.
Thank you for your consideration. Your participation is invaluable to the success of this study. We want to inform that we expect this survey to take ~10 minutes to complete.

Sincerely,
Mohamed A Mohamed

*

If you agree to participate, please tick the following option:

○ I consent to participate in this Delphi study

2. **My email address is** *

3. **Which professional group do you belong to?** *

Intensivist	ICU Dietician	ICU physiotherapist
○	○	○

Knowledge and confidence regarding muscle maintenance in the ICU

4. **I am confident prescribing protein nutrition for the ICU patients** *

Strongly disagree	Disagree	Neutral	Agree	Strongly agree
○	○	○	○	○

5. **I am aware of skeletal muscle mass assessment tools in the ICU environment** *

Strongly disagree	Disagree	Neutral	Agree	Strongly agree
○	○	○	○	○

6. **What skeletal muscle mass assessment tools in the ICU are you aware of?**

7. **I am aware of skeletal muscle function assessment tools in the ICU environment** *

Strongly disagree	Disagree	Neutral	Agree	Strongly agree
○	○	○	○	○

8. **What skeletal muscle function assessment tools in the ICU are you aware of?**

Opinions about protein intake in the ICU

9. **Protein dosage in the ICU should be:** *

	Strongly disagree	Disagree	Neutral	Agree	Strongly agree	Unsure
</=0.8 g/kg body weight	○	○	○	○	○	○
0.8-1.2 g/kg body weight	○	○	○	○	○	○
1.2 -1.5 g/kg body weight	○	○	○	○	○	○
>/=1.5 g/kg body weight	○	○	○	○	○	○

10. **Please add any comments to support your answer to the question above.**

11. **Optimal protein intake could impact patient-centred outcomes in the following ways:** *

	Strongly disagree	Disagree	Neutral	Agree	Strongly agree	Unsure
Increase muscle mass	○	○	○	○	○	○
Decrease muscle mass	○	○	○	○	○	○
Increase muscle function	○	○	○	○	○	○
Decrease muscle function	○	○	○	○	○	○

Mitigate muscle mass losses	◯	◯	◯	◯	◯	◯
Mitigate muscle function losses	◯	◯	◯	◯	◯	◯
Improve physical function following discharge	◯	◯	◯	◯	◯	◯
Worsen physical function following discharge	◯	◯	◯	◯	◯	◯
Improve quality of life following discharge	◯	◯	◯	◯	◯	◯
Worsen quality of life following discharge	◯	◯	◯	◯	◯	◯
Reduce risk of discharge to rehabilitation facilities	◯	◯	◯	◯	◯	◯
Increase risk of discharge to rehabilitation facilities	◯	◯	◯	◯	◯	◯

12. **Please add any comments to support your answer to the question above.**

13. **Optimal protein intake could impact conventional clinical outcomes in the following ways:** *

	Strongly disagree	Disagree	Neutral	Agree	Strongly agree	Unsure
Reduce ICU mortality	◯	◯	◯	◯	◯	◯
Increase ICU mortality	◯	◯	◯	◯	◯	◯
Shorten ICU length of stay	◯	◯	◯	◯	◯	◯
Lengthen ICU length of stay	◯	◯	◯	◯	◯	◯
Increase ventilator free days	◯	◯	◯	◯	◯	◯
Decrease ventilator free days	◯	◯	◯	◯	◯	◯
Decrease risk of sepsis	◯	◯	◯	◯	◯	◯
Increase risk of sepsis	◯	◯	◯	◯	◯	◯
Negatively impact renal function	◯	◯	◯	◯	◯	◯
Positively impact renal function	◯	◯	◯	◯	◯	◯
Enhance wound healing	◯	◯	◯	◯	◯	◯

Worsen wound healing	○	○	○	○	○	○

14. **Please add any comments to support your answer to the question above.**

15. **Following ICU admission protein nutrition should start:** *

	Strongly disagree	Disagree	Neutral	Agree	Strongly agree	Unsure
Within <24 hours	○	○	○	○	○	○
Within 24-48 hours	○	○	○	○	○	○
Within 48-72 hours	○	○	○	○	○	○
>72 hours	○	○	○	○	○	○

16. **Please add any comments to support your answer to the question above.**

17. **Early protein supplementation in the ICU could:** *

	Strongly disagree	Disagree	Neutral	Agree	Strongly agree	Unsure
Be harmful for septic patients	○	○	○	○	○	○
Be associated with less nutritional deficits	○	○	○	○	○	○
Improve patient-centred functional outcomes	○	○	○	○	○	○
Improve conventional clinical outcomes	○	○	○	○	○	○

18. **Please add any comments to support your answer to the question above.**

19. **Considering intermittent versus continuous protein provision in the ICU.** *

	Strongly disagree	Disagree	Neutral	Agree	Strongly agree	Unsure
Intermittent, compared to continuous, provision could enhance the cellular process of muscle building	○	○	○	○	○	○
Continuous, compared to intermittent, provision is more feasible in the ICU environment	○	○	○	○	○	○
Intermittent, compared to continuous, enteral provision could have a negative effect on gastric function (e.g., vomiting and aspirates)	○	○	○	○	○	○

20. **Please add any comments to support your answer to the question above.**

Opinions about adjuvant exercise in the ICU

21. **Exercise' (contractile activity) adjuvant to optimal protein intake could impact patient-centred functional outcomes in the following ways:** *

	Strongly disagree	Disagree	Neutral	Agree	Strongly agree	Unsure
Increase muscle mass	○	○	○	○	○	○
Decrease muscle mass	○	○	○	○	○	○
Increase muscle function	○	○	○	○	○	○
Decrease muscle function	○	○	○	○	○	○
Mitigate muscle mass losses	○	○	○	○	○	○
Mitigate muscle function losses	○	○	○	○	○	○
Improve physical function following discharge	○	○	○	○	○	○
Worsen physical function following discharge	○	○	○	○	○	○
Improve quality of life following discharge	○	○	○	○	○	○
Worsen quality of life following discharge	○	○	○	○	○	○

Reduce risk of discharge to rehabilitation facilities	○	○	○	○	○	○
Increase risk of discharge to rehabilitation facilities	○	○	○	○	○	○

22. **Please add any comments to support your answer to the question above.**

23. **Exercise' (contractile activity) adjuvant to optimal protein intake could impact conventional clinical outcomes in the following ways:** *

	Strongly disagree	Disagree	Neutral	Agree	Strongly agree	Unsure
Reduce ICU mortality	○	○	○	○	○	○
Increase ICU mortality	○	○	○	○	○	○
Shorten ICU length of stay	○	○	○	○	○	○
Increase ICU length of stay	○	○	○	○	○	○
Increase ventilator free days	○	○	○	○	○	○
Decrease ventilator free days	○	○	○	○	○	○

24. **Please add any comments to support your answer to the question above.**

This content is neither created nor endorsed by Microsoft. The data you submit will be sent to the form owner.

Microsoft Forms
