# Supplementary material for: Protein nutrition in the ICU: a Delphi exercise to highlight knowledge and opinions of different professional groups involved in patient critical care
Source: BMC Nutr. 2026 Apr 18;12:101. doi: 10.1186/s40795-026-01314-3 (PMC13220506; doi:10.1186/s40795-026-01314-3)
Supplement: Supplementary file 3 — Supplementary Material 3. [file 40795_2026_1314_MOESM3_ESM.pdf]

Round-2 Protein nutrition and adjuvant exercise in the ICU

second round of a Delphi exercise to highlight opinions of different professional groups involved in patient critical care

* Required

1. **My email address is** *

2. **Which professional group do you belong to?** *

Intensivist	ICU Dietician	ICU physiotherapist
○	○	○

Opinions about protein intake in the ICU

3. **Protein consumption in the ICU should be:** *

	Strongly Disagree	Disagree	Neutral	Agree	Strongly Agree	Unsure
</=0.8 g/kg body weight	◯	◯	◯	◯	◯	◯
0.8-1.2 g/kg body weight	◯	◯	◯	◯	◯	◯
1.2 -1.5 g/kg body weight	◯	◯	◯	◯	◯	◯
>/=1.5 g/kg body weight	◯	◯	◯	◯	◯	◯

4. **Adequate protein provision could affect patient-focused outcomes regarding:** *

	Strongly Disagree	Disagree	Neutral	Strongly Agree	Agree	Unsure
Increase muscle mass	◯	◯	◯	◯	◯	◯
Increase muscle function	◯	◯	◯	◯	◯	◯
Improve quality of life following discharge	◯	◯	◯	◯	◯	◯
Reduce risk of discharge to rehabilitation facilities	◯	◯	◯	◯	◯	◯
Increase risk of discharge to rehabilitation facilities	◯	◯	◯	◯	◯	◯
Mitigate muscle function loss	◯	◯	◯	◯	◯	◯
Improve physical function following discharge	◯	◯	◯	◯	◯	◯

5. **Adequate protein provision could affect standard clinical outcomes regarding:** *

	Strongly Disagree	Disagree	Neutral	Strongly Agree	Agree	Unsure
Reduce ICU mortality	◯	◯	◯	◯	◯	◯
Increase ICU mortality	◯	◯	◯	◯	◯	◯
Shorten ICU length of stay	◯	◯	◯	◯	◯	◯
Lengthen ICU length of stay	◯	◯	◯	◯	◯	◯
Increase ventilator free days	◯	◯	◯	◯	◯	◯
Decrease ventilator free days	◯	◯	◯	◯	◯	◯
Decrease risk of sepsis	◯	◯	◯	◯	◯	◯
Increase risk of sepsis	◯	◯	◯	◯	◯	◯

Negatively impact renal function	◯	◯	◯	◯	◯	◯
Positively impact renal function	◯	◯	◯	◯	◯	◯
Worsen wound healing	◯	◯	◯	◯	◯	◯

6. **Initiation of protein provision post ICU discharge could be:** *

	Strongly Disagree	Disagree	Neutral	Strongly Agree	Agree	Unsure
Within <24 hours	◯	◯	◯	◯	◯	◯
Within 24-48 hours	◯	◯	◯	◯	◯	◯
Within 48-72 hours	◯	◯	◯	◯	◯	◯
>72 hours	◯	◯	◯	◯	◯	◯

7. **Early amino acids supplementation in the ICU could:** *

	Strongly Disagree	Disagree	Neutral	Strongly Agree	Agree	Unsure
Adversely affect septic patients	○	○	○	○	○	○
Be correlated with reduced nutritional deficiencies	○	○	○	○	○	○
Improve patient-focused functional outcomes	○	○	○	○	○	○
Improve standard clinical outcomes	○	○	○	○	○	○

8. **Considering boluses of protein delivery versus steady state delivery in the ICU.** *

	Strongly Disagree	Disagree	Neutral	Strongly Agree	Agree	Unsure
boluses, compared to steady state delivery, could enhance the cellular process of muscle building	○	○	○	○	○	○
Steady state delivery, compared to boluses, is more feasible in the ICU environment	○	○	○	○	○	○

boluses, compared to steady state enteral delivery could have a negative effect on gastric function *(e.g., vomiting and aspirates)*	◯	◯	◯	◯	◯	◯

Opinions about adjuvant exercise in the ICU

9. **'Exercise' (contractile activity) concomitant to adequate protein intake could affect patient-focused outcomes regarding:** *

	Strongly Disagree	Disagree	Neutral	Strongly Agree	Agree	Unsure
Increase muscle mass	◯	◯	◯	◯	◯	◯
Reduce risk of discharge to rehabilitation facilities	◯	◯	◯	◯	◯	◯
Increase risk of discharge to rehabilitation facilities	◯	◯	◯	◯	◯	◯
Mitigate muscle function loss	◯	◯	◯	◯	◯	◯
Improve quality of life following discharge	◯	◯	◯	◯	◯	◯

10. **'Exercise' (contractile activity) concomitant to adequate protein intake could affect standard clinical outcomes regarding:** *

	Strongly Disagree	Disagree	Neutral	Strongly Agree	Agree	Unsure
Reduce ICU mortality	◯	◯	◯	◯	◯	◯
Increase ICU mortality	◯	◯	◯	◯	◯	◯
Shorten ICU length of stay	◯	◯	◯	◯	◯	◯
Increase ICU length of stay	◯	◯	◯	◯	◯	◯
Increase ventilator free days	◯	◯	◯	◯	◯	◯
Decrease ventilator free days	◯	◯	◯	◯	◯	◯

This content is neither created nor endorsed by Microsoft. The data you submit will be sent to the form owner.

Microsoft Forms
