# Supplementary material for: Protein nutrition in the ICU: a Delphi exercise to highlight knowledge and opinions of different professional groups involved in patient critical care
Source: BMC Nutr. 2026 Apr 18;12:101. doi: 10.1186/s40795-026-01314-3 (PMC13220506; doi:10.1186/s40795-026-01314-3)
Supplement: Supplementary file 4 — Supplementary Material 4. [file 40795_2026_1314_MOESM4_ESM.docx]

**Delphi study**

**Stability testing by Coefficient of variation**

Protein Intake Dosage Recommendations (Round-1 All Participants)

Results: Coefficient of Variation (CV) by Protein Intake Category

| Category | Mean (μ) | Standard Deviation (σ) | CV (%) |  |
| --- | --- | --- | --- | --- |
| ≤0.8 g/kg | 1.33 | 0.69 | 51.7% |  |
| 0.8–1.2 g/kg | 2.32 | 1.02 | 44.0% |  |
| 1.2–1.5 g/kg | 2.78 | 0.58 | 21.0% |  |
| ≥1.5 g/kg | 2.02 | 0.91 | 44.8% |  |

Protein Intake Dosage Recommendations (Round-2 All Participants)

Results: Coefficient of Variation (CV) by Protein Intake Category

| Category | Mean (μ) | Standard Deviation (σ) | CV (%) |  |
| --- | --- | --- | --- | --- |
| ≤0.8 g/kg | 1.19 | 0.54 | 45.4% |  |
| 0.8–1.2 g/kg | 2.08 | 0.82 | 39.5% |  |
| 1.2–1.5 g/kg | 2.42 | 0.66 | 27.3% |  |
| ≥1.5 g/kg | 1.85 | 0.93 | 50.3% |  |

Protein Intake Dosage Recommendations (Round-3 All Participants)

Results: Coefficient of Variation (CV) by Protein Intake Category

| Category | Mean (μ) | Standard Deviation (σ) | CV (%) |  |
| --- | --- | --- | --- | --- |
| ≤0.8 g/kg | 1.33 | 0.57 | 0.43 |  |
| 0.8–1.2 g/kg | 2.27 | 0.76 | 0.33 |  |
| 1.2–1.5 g/kg | 2.74 | 0.60 | 0.22 |  |
| ≥1.5 g/kg | 1.70 | 0.78 | 0.46 |  |

Protein Intake Initiation Recommendations (Round-1 All Participants)

Results: Coefficient of Variation (CV) by Timing Category

| Category | Mean (μ) | Standard Deviation (σ) | CV (%) |  |
| --- | --- | --- | --- | --- |
| Within <24 hours | 1.93 | 0.96 | 49.7% |  |
| 24–48 hours | 2.64 | 0.72 | 27.3% |  |
| 48–72 hours | 2.07 | 0.97 | 46.9% |  |
| >72 hours | 1.67 | 0.95 | 56.9% |  |

Protein Intake Initiation Recommendations (Round-2 All Participants)

Results: Coefficient of Variation (CV) by Timing Category

| Category | Mean (μ) | Standard Deviation (σ) | CV (%) |  |
| --- | --- | --- | --- | --- |
| Within <24 hours | 1.85 | 0.86 | 46.5% |  |
| 24–48 hours | 2.35 | 0.70 | 29.8% |  |
| 48–72 hours | 2.10 | 0.85 | 40.5% |  |
| >72 hours | 1.88 | 0.94 | 50.0% |  |

Protein Intake Initiation Recommendations (Round-3 All Participants)

Results: Coefficient of Variation (CV) by Timing Category

| Category | Mean (μ) | Standard Deviation (σ) | CV (%) |  |
| --- | --- | --- | --- | --- |
| Within <24 hours | 1.28 | 0.63 | 0.49 |  |
| 24–48 hours | 1.68 | 0.86 | 0.51 |  |
| 48–72 hours | 2.18 | 0.85 | 0.39 |  |
| >72 hours | 2.47 | 0.78 | 0.28 |  |

Patient-centred outcome category (Round-1 All Participants)

Results: Coefficient of Variation (CV) by patient-centred outcome category

| Item | Mean | SD | CV (%) |  |
| --- | --- | --- | --- | --- |
| Increase Muscle Mass | 2.15 | 0.83 | 38.6% |  |
| Decrease Muscle Mass | 1.12 | 0.34 | 30.4% |  |
| Increase Muscle Function | 2.62 | 0.71 | 27.1% |  |
| Decrease Muscle Function | 1.08 | 0.28 | 25.9% |  |
| Mitigate Muscle Mass Losses | 2.89 | 0.42 | 14.5% |  |
| Mitigate Muscle Function Losses | 2.75 | 0.54 | 19.6% |  |
| Improve Physical Function Post-Discharge | 2.81 | 0.49 | 17.4% |  |
| Worsen Physical Function Post-Discharge | 1.18 | 0.41 | 34.7% |  |
| Improve Quality of Life Post-Discharge | 2.67 | 0.63 | 23.6% |  |
| Worsen Quality of Life Post-Discharge | 1.09 | 0.31 | 28.4% |  |
| Reduce Risk of Discharge to Rehab | 1.89 | 0.77 | 40.7% |  |
| Increase Risk of Discharge to Rehab | 1.45 | 0.62 | 42.8% |  |

Patient-centred outcome category (Round-2 All Participants)

Results: Coefficient of Variation (CV) by patient-centred outcome category

| Outcome | Mean | SD | CV (%) |  |
| --- | --- | --- | --- | --- |
| Increase Muscle Mass | 2.15 | 0.83 | 38.6% |  |
| Increase Muscle Function | 2.20 | 0.85 | 38.6% |  |
| Mitigate Muscle Function Losses | 2.78 | 0.42 | 15.1% |  |
| Improve Physical Function Post-Discharge | 2.65 | 0.54 | 20.4% |  |
| Improve Quality of Life Post-Discharge | 2.60 | 0.63 | 24.2% |  |
| Reduce Risk of Discharge to Rehab | 1.92 | 0.77 | 40.1% |  |
| Increase Risk of Discharge to Rehab | 1.18 | 0.41 | 34.7% |  |

Patient-centred outcome category (Round-3 All Participants)

Results: Coefficient of Variation (CV) by patient-centred outcome category

| Outcome | Mean | SD | CV (%) |  |
| --- | --- | --- | --- | --- |
| Increase Muscle Mass | 2.3 | 0.78 | 0.34 |  |
| Increase Muscle Function | 2.48 | 0.74 | 0.30 |  |
| Improve QoL Post-Discharge | 2.71 | 0.55 | 0.20 |  |
| Reduce Risk of Discharge to Rehab | 2.69 | 0.60 | 0.22 |  |
| Increase Risk of Discharge to Rehab | 1.51 | 0.81 | 0.54 |  |

Conventional outcome category (Round-1 All Participants)

Results: Coefficient of Variation (CV) by conventional outcome category

| Outcome | Mean | SD | CV (%) |
| --- | --- | --- | --- |
| Reduce ICU Mortality | 2.72 | 0.63 | 23.2% |
| Increase ICU Mortality | 1.15 | 0.43 | 37.4% |
| Shorten ICU LOS | 2.58 | 0.72 | 27.9% |
| Lengthen ICU LOS | 1.18 | 0.49 | 41.5% |
| Increase Ventilator-Free Days | 2.51 | 0.76 | 30.3% |
| Decrease Ventilator-Free Days | 1.12 | 0.38 | 33.9% |
| Decrease Sepsis Risk | 2.34 | 0.81 | 34.6% |
| Increase Sepsis Risk | 1.08 | 0.31 | 28.7% |
| Negatively Impact Renal Function | 1.62 | 0.85 | 52.5% |
| Positively Impact Renal Function | 1.89 | 0.78 | 41.3% |
| Enhance Wound Healing | 2.93 | 0.32 | 10.9% |
| Worsen Wound Healing | 1.05 | 0.28 | 26.7% |

Conventional outcome category (Round-2 All Participants)

Results: Coefficient of Variation (CV) by conventional outcome category

| Outcome | Mean | SD | CV (%) |
| --- | --- | --- | --- |
| Reduce ICU Mortality | 2.12 | 0.89 | 42.0% |
| Increase ICU Mortality | 1.08 | 0.31 | 28.7% |
| Shorten ICU Length of Stay | 2.18 | 0.85 | 39.0% |
| Lengthen ICU Length of Stay | 1.15 | 0.43 | 37.4% |
| Increase Ventilator-Free Days | 2.10 | 0.82 | 39.0% |
| Decrease Ventilator-Free Days | 1.14 | 0.43 | 37.7% |
| Decrease Sepsis Risk | 1.92 | 0.91 | 47.4% |
| Increase Sepsis Risk | 1.08 | 0.31 | 28.7% |
| Negatively Impact Renal Function | 1.41 | 0.64 | 45.4% |
| Positively Impact Renal Function | 1.71 | 0.81 | 47.4% |

Conventional outcome category (Round-3 All Participants)

Results: Coefficient of Variation (CV) by conventional outcome category

| Outcome | Mean | SD | CV (%) |
| --- | --- | --- | --- |
| Reduce ICU Mortality | 2.40 | 0.71 | 0.30 |
| Increase ICU Mortality | 1.26 | 0.59 | 0.47 |
| Shorten ICU Length of Stay | 2.54 | 0.64 | 0.25 |
| Lengthen ICU Length of Stay | 1.35 | 0.69 | 0.51 |
| Increase Ventilator-Free Days | 2.45 | 0.72 | 0.29 |
| Decrease Ventilator-Free Days | 1.25 | 0.58 | 0.46 |
| Decrease Sepsis Risk | 2.14 | 0.68 | 0.32 |
| Increase Sepsis Risk | 1.34 | 0.47 | 0.35 |
| Negatively Impact Renal Function | 1.78 | 0.77 | 0.43 |
| Positively Impact Renal Function | 1.94 | 0.70 | 0.36 |

Effects of early protein supplementation in the ICU category (Round-1 All Participants)

| Outcome | Mean | SD | CV (%) |
| --- | --- | --- | --- |
| Be harmful for septic patients | 1.82 | 0.98 | 53.8% |
| Associated with less nutritional deficits | 2.72 | 0.63 | 23.2% |
| Improve patient-centred functional outcomes | 2.85 | 0.53 | 18.6% |
| Improve conventional clinical outcomes | 2.70 | 0.67 | 24.8% |

Effects of early protein supplementation in the ICU category (Round-2 All Participants)

| Outcome | Mean | SD | CV (%) |
| --- | --- | --- | --- |
| Be harmful for septic patients | 1.87 | 0.97 | 51.9% |
| Associated with less nutritional deficits | 2.48 | 0.73 | 29.4% |
| Improve patient-centred functional outcomes | 2.53 | 0.79 | 31.2% |
| Improve conventional clinical outcomes | 2.36 | 0.84 | 35.6% |

Effects of early protein supplementation in the ICU category (Round-3 All Participants)

| Outcome | Mean | SD | CV (%) |
| --- | --- | --- | --- |
| Be harmful for septic patients | 2.02 | 0.79 | 0.39 |
| Associated with less nutritional deficits | 2.38 | 0.71 | 0.30 |
| Improve patient-centred functional outcomes | 2.36 | 0.73 | 0.31 |
| Improve conventional clinical outcomes | 2.27 | 0.78 | 0.34 |

intermittent versus continuous protein provision in the ICU category (Round-1 All Participants)

| Outcome | Mean | SD | CV (%) |
| --- | --- | --- | --- |
| Intermittent, compared to continuous, provision could enhance the cellular process of muscle building | 2.30 | 0.62 | 27.0 |
| Continuous, compared to intermittent, provision is more feasible in the ICU environment | 2.45 | 0.77 | 31.4 |
| Intermittent, compared to continuous, enteral provision could have a negative effect on gastric function (e.g., vomiting and aspirates) | 1.98 | 0.71 | 36.0 |

intermittent versus continuous protein provision in the ICU category (Round-2 All Participants)

| Outcome | Mean | SD | CV (%) |
| --- | --- | --- | --- |
| Intermittent, compared to continuous, provision could enhance the cellular process of muscle building | 2.39 | 0.71 | 29.7 |
| Continuous, compared to intermittent, provision is more feasible in the ICU environment | 2.53 | 0.73 | 28.8 |
| Intermittent, compared to continuous, enteral provision could have a negative effect on gastric function (e.g., vomiting and aspirates) | 2.11 | 0.74 | 35.0 |

intermittent versus continuous protein provision in the ICU category (Round-3 All Participants)

| Outcome | Mean | SD | CV (%) |
| --- | --- | --- | --- |
| Intermittent, compared to continuous, provision could enhance the cellular process of muscle building | 2.17 | 0.65 | 0.30 |
| Continuous, compared to intermittent, provision is more feasible in the ICU environment | 2.55 | 0.65 | 0.25 |
| Intermittent, compared to continuous, enteral provision could have a negative effect on gastric function (e.g., vomiting and aspirates) | 2.29 | 0.73 | 0.32 |

Exercise’ (contractile activity) adjuvant to optimal protein intake could impact patient-centred functional outcomes category (Round-1 All Participants)

| Item | Mean | SD | CV (%) |
| --- | --- | --- | --- |
| Increase muscle mass | 2.73 | 0.58 | 21.2 |
| Decrease muscle mass | 1.12 | 0.37 | 33.0 |
| Increase muscle function | 2.95 | 0.22 | 7.5 |
| Decrease muscle function | 1.08 | 0.28 | 25.9 |
| Mitigate muscle mass losses | 2.82 | 0.43 | 15.2 |
| Mitigate muscle function losses | 2.75 | 0.53 | 19.3 |
| Improve physical function following discharge | 2.89 | 0.34 | 11.8 |
| Worsen physical function following discharge | 1.15 | 0.42 | 36.5 |
| Improve quality of life following discharge | 2.67 | 0.61 | 22.8 |
| Worsen quality of life following discharge | 1.10 | 0.35 | 31.8 |
| Reduce risk of discharge to rehabilitation | 2.45 | 0.78 | 31.8 |
| Increase risk of discharge to rehabilitation | 1.20 | 0.45 | 37.5 |

Exercise’ (contractile activity) adjuvant to optimal protein intake could impact patient-centred functional outcomes category (Round-2 All Participants)

| Item | Mean | SD | CV (%) |
| --- | --- | --- | --- |
| Increase muscle mass | 2.44 | 0.77 | 31.6 |
| Mitigate muscle function losses | 2.86 | 0.45 | 15.8 |
| Improve quality of life following discharge | 2.82 | 0.49 | 17.2 |
| Reduce risk of discharge to rehabilitation | 2.73 | 0.57 | 21.0 |
| Increase risk of discharge to rehabilitation | 1.45 | 0.74 | 50.9 |

Exercise’ (contractile activity) adjuvant to optimal protein intake could impact patient-centred functional outcomes category (Round-3 All Participants)

| Item | Mean | SD | CV (%) |
| --- | --- | --- | --- |
| Increase muscle mass | 2.38 | 0.80 | 0.34 |
| Reduce risk of discharge to rehab | 2.69 | 0.51 | 0.19 |
| Increase risk of discharge to rehab | 1.31 | 0.61 | 0.47 |

Exercise’ (contractile activity) adjuvant to optimal protein intake could impact conventional outcomes category (Round-1 All Participants)

| Item | Mean | SD | CV (%) |
| --- | --- | --- | --- |
| Reduce ICU mortality | 2.50 | 0.67 | 26.6 |
| Increase ICU mortality | 1.32 | 0.57 | 43.1 |
| Shorten ICU length of stay | 2.75 | 0.54 | 19.5 |
| Increase ICU length of stay | 1.25 | 0.54 | 43.2 |
| Increase ventilator-free days | 2.71 | 0.57 | 21.0 |
| Decrease ventilator-free days | 1.29 | 0.58 | 45.0 |

Exercise’ (contractile activity) adjuvant to optimal protein intake could impact conventional outcomes category (Round-2 All Participants)

| Item | Mean | SD | CV (%) |
| --- | --- | --- | --- |
| Reduce ICU mortality | 2.48 | 0.67 | 0.27 |
| Increase ICU mortality | 1.23 | 0.42 | 0.35 |
| Shorten ICU length of stay | 2.59 | 0.59 | 0.23 |
| Increase ICU length of stay | 1.20 | 0.4 | 0.34 |
| Increase ventilator-free days | 2.33 | 0.81 | 0.35 |
| Decrease ventilator-free days | 1.4 | 0.66 | 0.47 |

Exercise’ (contractile activity) adjuvant to optimal protein intake could impact conventional outcomes category (Round-3 All Participants)

|  |  |  | |  | |  |  |
| --- | --- | --- | --- | --- | --- | --- | --- |
| Item | | | Mean | | SD | | CV (%) |
| Reduce ICU mortality | | | 2.35 | | 0.74 | | 0.31 |
| Increase ICU mortality | | | 1.26 | | 0.59 | | 0.47 |
| Shorten ICU length of stay | | | 2.65 | | 0.61 | | 0.23 |
| Increase ICU length of stay | | | 1.2 | | 0.46 | | 0.38 |
| Increase ventilator-free days | | | 2.63 | | 0.58 | | 0.22 |
| Decrease ventilator-free days | | | 1.21 | | 0.40 | | 0.33 |

Absolute CV differences across Delphi rounds

| Outcome | Round-1 CV% | Round-2 CV% | Round-3 CV% | CV (R1-R2) | CV (R2-R3) |  |
| --- | --- | --- | --- | --- | --- | --- |
| Protein Intake Dosage |  | | |  |  |  |
| </= 0.8 g.kg.d | 51.7% | 45.4% | 0.43 | \| 6.30% \| \| --- \| | 0.02 |  |
| 0.8-1.2 g.kg.d | 44.0% | 39.5% | 0.33 | 4.5% | 0.06 |  |
| 1.2-1.5 g.kg.d | 21.0% | 27.3% | 0.22 | -6.30% | 0.05 |  |
| >/= 1.5 g.kg.d | 44.8% | 50.3% | 0.46 | \| -5.50% \| \| --- \| | 0.04 |  |
| Protein Intake Initiation |  |  |  |  |  |  |
| < 24 hrs. | 49.7% | 46.5% | 0.49 | \| 3.20% \| \| --- \| | -0.03 |  |
| 24-48 hrs. | 27.3% | 29.8% | 0.51 | -2.5% | -0.21 |  |
| 48-72 hrs. | 46.9% | 40.5% | 0.39 | 6.4% | 0.01 |  |
| 72 hrs. | 56.9% | 50.0% | 0.28 | 6.9% | 0.22 |  |
| Patient-centred outcome |  |  |  |  |  |  |
| Increase Muscle Mass | 38.6% | 38.6% | 0.34 | 0% | 0.04 |  |
| Decrease Muscle Mass | 30.4% |  |  |  |  |  |
| Increase Muscle Function | 27.1% | 38.6% | 0.30. | -11.5% | 0.08 |  |
| Decrease Muscle Function | 25.9% |  |  |  |  |  |
| Mitigate Muscle Mass Losses | 14.5% |  |  |  |  |  |
| Mitigate Muscle Function Losses | 19.6% | 15.1% |  | 4.6% |  |  |
| Improve Physical Function Post-Discharge | 17.4% | 20.4% |  | -3% |  |  |
| Worsen Physical Function Post-Discharge | 34.7% |  |  |  |  |  |
| Improve Quality of Life Post-Discharge | 23.6% | 24.2% | 0.20 | -0.6% | 0.04 |  |
| Worsen Quality of Life Post-Discharge | 28.4% |  |  |  |  |  |
| Reduce Risk of Discharge to Rehab | 40.7% | 40.1% | 0.22 | 0.6% | 0.18 |  |
| Increase Risk of Discharge to Rehab | 42.8% | 34.7% | 0.54 | 8.1% | -0.2 |  |
| Conventional outcome |  |  |  |  |  |  |
| Reduce ICU Mortality | 23.2% | 42.0% | 0.30 | \| -18.80% \| \| --- \| | 0.12 |  |
| Increase ICU Mortality | 37.4% | 28.7% | 0.47 | 8.7% | -0.19 |  |
| Shorten ICU LOS | 27.9% | 39.0% | 0.25 | -11.1% | 0.14 |  |
| Lengthen ICU LOS | 41.5% | 37.4% | 0.51 | 4.1% | -0.14 |  |
| Increase Ventilator-Free Days | 30.3% | 39.0% | 0.29 | -8.7% | 0.1 |  |
| Decrease Ventilator-Free Days | 33.9% | 37.7% | 0.46 | -3.8% | -0.09 |  |
| Decrease Sepsis Risk | 34.6% | 47.4% | 0.32 | -12.8% | 0.15 |  |
| Increase Sepsis Risk | 28.7% | 28.7% | 0.35 | 0% | -0.07 |  |
| Negatively Impact Renal Function | 52.5% | 45.4% | 0.43 | 7.1% | 0.02 |  |
| Positively Impact Renal Function | 41.3% | 47.4% | 0.36 | -6.1% | 0.11 |  |
| Enhance Wound Healing | 10.9% |  |  |  |  |  |
| Worsen Wound Healing | 26.7% |  |  |  |  |  |
| Effects of early protein supplementation in the IC |  |  |  |  |  |  |
| Be harmful for septic patients | 53.8% | 51.9% | 0.39 | \| 1.90% \| \| --- \| | 0.13 |  |
| Associated with less nutritional deficits | 23.2% | 29.4% | 0.30 | -6.2% | -0.01 |  |
| Improve patient-centred functional outcomes | 18.6% | 31.2% | 0.31 | -12.6% | 0 |  |
| Improve conventional clinical outcomes | 24.8% | 35.6% | 0.34 | -10.8% | 0.01 |  |
| intermittent versus continuous protein provision in the ICU |  |  |  |  |  |  |
| Intermittent, compared to continuous, provision could enhance the cellular process of muscle building | 27.0% | 29.7% | 0.30 | \| -2.70% \| \| --- \| | -0.01 |  |
| Continuous, compared to intermittent, provision is more feasible in the ICU environment | 31.4% | 28.8% | 0.25 | 2.6% | 0.04 |  |
| Intermittent, compared to continuous, enteral provision could have a negative effect on gastric function (e.g., vomiting and aspirates) | 36.0% | 35.0% | 0.32 | 1% | 0.03 |  |
| Exercise’ (contractile activity) adjuvant to optimal protein intake could impact patient-centred functional outcomes |  |  |  |  |  |  |
| Increase muscle mass | 21.2% | 31.6% | 0.34 | -10.4% | -0.02 |  |
| Decrease muscle mass | 33.0% |  |  |  |  |  |
| Increase muscle function | 7.5% |  |  |  |  |  |
| Decrease muscle function | 25.9% |  |  |  |  |  |
| Mitigate muscle mass losses | 15.2% |  |  |  |  |  |
| Mitigate muscle function losses | 19.3% | 15.8% |  | 3.5% |  |  |
| Improve physical function following discharge | 11.8% |  |  |  |  |  |
| Worsen physical function following discharge | 36.5% |  |  |  |  |  |
| Improve quality of life following discharge | 22.8% | 17.2% |  | 5.6% |  |  |
| Worsen quality of life following discharge | 31.8% |  |  |  |  |  |
| Reduce risk of discharge to rehabilitation | 31.8% | 21.0% | 0.19 | 10.8% | 0.02 |  |
| Increase risk of discharge to rehabilitation | 37.5% | 50.9% | 0.47 | -13.4% | 0.03 |  |
| Exercise’ (contractile activity) adjuvant to optimal protein intake could impact conventional outcomes |  |  |  |  |  |  |
| Reduce ICU mortality | 26.6% | 27% | 0.31 | -0.4% | -0.04 |  |
| Increase ICU mortality | 43.1% | 35% | 0.47 | 8.1% | -0.12 |  |
| Shorten ICU length of stay | 19.5% | 23% | 0.23 | -3.5% | 0 |  |
| Increase ICU length of stay | 43.2% | 34% | 0.38 | 9.2% | -0.04 |  |
| Increase ventilator-free days | 21.0% | 35% | 0.22 | -14% | 0.13 |  |
| Decrease ventilator-free days | 45.0% | 47% | 0.33 | -2% | 0.14 |  |
