# Supplementary material for: Protein nutrition in the ICU: a Delphi exercise to highlight knowledge and opinions of different professional groups involved in patient critical care
Source: BMC Nutr. 2026 Apr 18;12:101. doi: 10.1186/s40795-026-01314-3 (PMC13220506; doi:10.1186/s40795-026-01314-3)
Supplement: Supplementary file 5 — Supplementary Material 5. [file 40795_2026_1314_MOESM5_ESM.pdf]

Delphi study

Stability testing by Coefficient of variation

Protein Intake Dosage Recommendations (Round-1 All Participants)

Results: Coefficient of Variation (CV) by Protein Intake Category

Category	Mean (μ)	Standard Deviation (σ)	CV (%)
≤0.8 g/kg	1.33	0.69	51.7%
0.8–1.2 g/kg	2.32	1.02	44.0%
1.2–1.5 g/kg	2.78	0.58	21.0%
≥1.5 g/kg	2.02	0.91	44.8%

Protein Intake Dosage Recommendations (Round-2 All Participants)

Results: Coefficient of Variation (CV) by Protein Intake Category

Category	Mean (μ)	Standard Deviation (σ)	CV (%)
≤0.8 g/kg	1.19	0.54	45.4%
0.8–1.2 g/kg	2.08	0.82	39.5%
1.2–1.5 g/kg	2.42	0.66	27.3%
≥1.5 g/kg	1.85	0.93	50.3%

Protein Intake Dosage Recommendations (Round-3 All Participants)

Results: Coefficient of Variation (CV) by Protein Intake Category

Category	Mean (μ)	Standard Deviation (σ)	CV (%)
≤0.8 g/kg	1.41	0.61	43%
0.8–1.2 g/kg	2.24	0.78	34.8%
1.2–1.5 g/kg	2.7	0.63	23.3%
≥1.5 g/kg	1.71	0.77	45%

Protein Intake Initiation Recommendations (Round-1 All Participants)

Results: Coefficient of Variation (CV) by Timing Category

Category	Mean (μ)	Standard Deviation (σ)	CV (%)
Within <24 hours	1.93	0.96	49.7%
24–48 hours	2.64	0.72	27.3%
48–72 hours	2.07	0.97	46.9%
>72 hours	1.67	0.95	56.9%

Protein Intake Initiation Recommendations (Round-2 All Participants)

Results: Coefficient of Variation (CV) by Timing Category

Category	Mean (μ)	Standard Deviation (σ)	CV (%)
Within <24 hours	1.85	0.86	46.5%
24–48 hours	2.35	0.70	29.8%
48–72 hours	2.10	0.85	40.5%
>72 hours	1.88	0.94	50.0%

Protein Intake Initiation Recommendations (Round-3 All Participants)

Results: Coefficient of Variation (CV) by Timing Category

Category	Mean (μ)	Standard Deviation (σ)	CV (%)
Within <24 hours	1.06	0.33	31.1%
24–48 hours	1.40	0.83	59.3%
48–72 hours	2.54	0.75	29.5%
>72 hours	1.47	0.76	51.7%

Patient-centred outcome category (Round-1 All Participants)

Results: Coefficient of Variation (CV) by patient-centred outcome category

Item	Mean	SD	CV (%)
Increase Muscle Mass	2.15	0.83	38.6%
Decrease Muscle Mass	1.12	0.34	30.4%
Increase Muscle Function	2.62	0.71	27.1%
Decrease Muscle Function	1.08	0.28	25.9%
Mitigate Muscle Mass Losses	2.89	0.42	14.5%
Mitigate Muscle Function Losses	2.75	0.54	19.6%
Improve Physical Function Post-Discharge	2.81	0.49	17.4%
Worsen Physical Function Post-Discharge	1.18	0.41	34.7%
Improve Quality of Life Post-Discharge	2.67	0.63	23.6%
Worsen Quality of Life Post-Discharge	1.09	0.31	28.4%
Reduce Risk of Discharge to Rehab	1.89	0.77	40.7%
Increase Risk of Discharge to Rehab	1.45	0.62	42.8%

Patient-centred outcome category (Round-2 All Participants)

Results: Coefficient of Variation (CV) by patient-centred outcome category

Outcome	Mean	SD	CV (%)
Increase Muscle Mass	2.15	0.83	38.6%
Increase Muscle Function	2.20	0.85	38.6%
Mitigate Muscle Function Losses	2.78	0.42	15.1%
Improve Physical Function Post-Discharge	2.65	0.54	20.4%
Improve Quality of Life Post-Discharge	2.60	0.63	24.2%
Reduce Risk of Discharge to Rehab	1.92	0.77	40.1%
Increase Risk of Discharge to Rehab	1.18	0.41	34.7%

Patient-centred outcome category (Round-3 All Participants)

Results: Coefficient of Variation (CV) by patient-centred outcome category

Outcome	Mean	SD	CV (%)
Increase Muscle Mass	2.28	0.78	34.3%
Increase Muscle Function	2.47	0.75	30.5%
Improve QoL Post-Discharge	2.74	0.55	20%
Reduce Risk of Discharge to Rehab	2.66	0.62	23.3%
Increase Risk of Discharge to Rehab	1.79	0.87	48.6%

Conventional outcome category (Round-1 All Participants)

Results: Coefficient of Variation (CV) by conventional outcome category

Outcome	Mean	SD	CV (%)
Reduce ICU Mortality	2.72	0.63	23.2%
Increase ICU Mortality	1.15	0.43	37.4%
Shorten ICU LOS	2.58	0.72	27.9%
Lengthen ICU LOS	1.18	0.49	41.5%
Increase Ventilator-Free Days	2.51	0.76	30.3%
Decrease Ventilator-Free Days	1.12	0.38	33.9%
Decrease Sepsis Risk	2.34	0.81	34.6%
Increase Sepsis Risk	1.08	0.31	28.7%
Negatively Impact Renal Function	1.62	0.85	52.5%
Positively Impact Renal Function	1.89	0.78	41.3%
Enhance Wound Healing	2.93	0.32	10.9%
Worsen Wound Healing	1.05	0.28	26.7%

Conventional outcome category (Round-2 All Participants)

Results: Coefficient of Variation (CV) by conventional outcome category

Outcome	Mean	SD	CV (%)
Reduce ICU Mortality	2.12	0.89	42.0%
Increase ICU Mortality	1.08	0.31	28.7%
Shorten ICU Length of Stay	2.18	0.85	39.0%
Lengthen ICU Length of Stay	1.15	0.43	37.4%

Outcome	Mean	SD	CV (%)
Increase Ventilator-Free Days	2.10	0.82	39.0%
Decrease Ventilator-Free Days	1.14	0.43	37.7%
Decrease Sepsis Risk	1.92	0.91	47.4%
Increase Sepsis Risk	1.08	0.31	28.7%
Negatively Impact Renal Function	1.41	0.64	45.4%
Positively Impact Renal Function	1.71	0.81	47.4%

Conventional outcome category (Round-3 All Participants)

Results: Coefficient of Variation (CV) by conventional outcome category

Outcome	Mean	SD	CV (%)
Reduce ICU Mortality	2.44	0.72	29.6%
Increase ICU Mortality	1.27	0.60	47.2%
Shorten ICU Length of Stay	2.25	0.92	41.1%
Lengthen ICU Length of Stay	1.53	0.82	53.6%
Increase Ventilator-Free Days	1.22	0.62	50.8%
Decrease Ventilator-Free Days	2.58	0.75	29.1%
Decrease Sepsis Risk	2.15	0.89	41.4%
Increase Sepsis Risk	1.18	0.53	44.9%
Negatively Impact Renal Function	1.65	0.78	47.3%
Positively Impact Renal Function	2.03	0.85	41.9%

Effects of early protein supplementation in the ICU category (Round-1 All Participants)

Outcome	Mean	SD	CV (%)
Be harmful for septic patients	1.82	0.98	53.8%
Associated with less nutritional deficits	2.72	0.63	23.2%
Improve patient-centred functional outcomes	2.85	0.53	18.6%
Improve conventional clinical outcomes	2.70	0.67	24.8%

Effects of early protein supplementation in the ICU category (Round-2 All Participants)

Outcome	Mean	SD	CV (%)
Be harmful for septic patients	1.87	0.97	51.9%
Associated with less nutritional deficits	2.48	0.73	29.4%
Improve patient-centred functional outcomes	2.53	0.79	31.2%
Improve conventional clinical outcomes	2.36	0.84	35.6%

Effects of early protein supplementation in the ICU category (Round-3 All Participants)

Outcome	Mean	SD	CV (%)
Be harmful for septic patients	1.94	0.86	44.3%
Associated with less nutritional deficits	2.15	0.89	41.4%

Outcome	Mean	SD	CV (%)
Improve patient-centred functional outcomes	2.28	0.97	42.5%
Improve conventional clinical outcomes	2.12	0.95	44.8%

intermittent versus continuous protein provision in the ICU category (Round-1 All Participants)

Outcome	Mean	SD	CV (%)
Intermittent, compared to continuous, provision could enhance the cellular process of muscle building	2.30	0.62	27.0%
Continuous, compared to intermittent, provision is more feasible in the ICU environment	2.45	0.77	31.4%
Intermittent, compared to continuous, enteral provision could have a negative effect on gastric function (e.g., vomiting and aspirates)	1.98	0.71	36.0%

intermittent versus continuous protein provision in the ICU category (Round-2 All Participants)

Outcome	Mean	SD	CV (%)
Intermittent, compared to continuous, provision could enhance the cellular process of muscle building	2.39	0.71	29.7%
Continuous, compared to intermittent, provision is more feasible in the ICU environment	2.53	0.73	28.8%
Intermittent, compared to continuous, enteral provision could have a negative effect on gastric function (e.g., vomiting and aspirates)	2.11	0.74	35.0%

intermittent versus continuous protein provision in the ICU category (Round-3 All Participants)

Outcome	Mean	SD	CV (%)
Intermittent, compared to continuous, provision could enhance the cellular process of muscle building	2.17	0.65	29.8%
Continuous, compared to intermittent, provision is more feasible in the ICU environment	2.65	0.65	24.7%
Intermittent, compared to continuous, enteral provision could have a negative effect on gastric function (e.g., vomiting and aspirates)	2.29	0.73	31.8%

Exercise’ (contractile activity) adjuvant to optimal protein intake could impact patient-centred functional outcomes category (Round-1 All Participants)

Item	Mean	SD	CV (%)
Increase muscle mass	2.73	0.58	21.2%
Decrease muscle mass	1.12	0.37	33.0%
Increase muscle function	2.95	0.22	7.5%
Decrease muscle function	1.08	0.28	25.9%
Mitigate muscle mass losses	2.82	0.43	15.2%
Mitigate muscle function losses	2.75	0.53	19.3%
Improve physical function following discharge	2.89	0.34	11.8%
Worsen physical function following discharge	1.15	0.42	36.5%
Improve quality of life following discharge	2.67	0.61	22.8%
Worsen quality of life following discharge	1.10	0.35	31.8%

Item	Mean	SD	CV (%)
Reduce risk of discharge to rehabilitation	2.45	0.78	31.8%
Increase risk of discharge to rehabilitation	1.20	0.45	37.5%

Exercise' (contractile activity) adjuvant to optimal protein intake could impact patient-centred functional outcomes category (Round-2 All Participants)

Item	Mean	SD	CV (%)
Increase muscle mass	2.44	0.77	31.6%
Mitigate muscle function losses	2.86	0.45	15.8%
Improve quality of life following discharge	2.82	0.49	17.2%
Reduce risk of discharge to rehabilitation	2.73	0.57	21.0%
Increase risk of discharge to rehabilitation	1.45	0.74	50.9%

Exercise' (contractile activity) adjuvant to optimal protein intake could impact patient-centred functional outcomes category (Round-3 All Participants)

Item	Mean	SD	CV (%)
Increase muscle mass	2.38	0.80	33.5%
Reduce risk of discharge to rehab	2.72	0.50	18.5%
Increase risk of discharge to rehab	1.31	0.61	46.2%

Exercise' (contractile activity) adjuvant to optimal protein intake could impact conventional outcomes category (Round-1 All Participants)

Item	Mean	SD	CV (%)
Reduce ICU mortality	2.50	0.67	26.6%
Increase ICU mortality	1.32	0.57	43.1%
Shorten ICU length of stay	2.75	0.54	19.5%
Increase ICU length of stay	1.25	0.54	43.2%
Increase ventilator-free days	2.71	0.57	21.0%
Decrease ventilator-free days	1.29	0.58	45.0%

Exercise' (contractile activity) adjuvant to optimal protein intake could impact conventional outcomes category (Round-2 All Participants)

Item	Mean	SD	CV (%)
Reduce ICU mortality	2.48	0.67	27%
Increase ICU mortality	1.23	0.42	35%
Shorten ICU length of stay	2.59	0.59	23%
Increase ICU length of stay	1.20	0.4	34%
Increase ventilator-free days	2.33	0.81	35%
Decrease ventilator-free days	1.4	0.66	47%

Exercise' (contractile activity) adjuvant to optimal protein intake could impact conventional outcomes category (Round-3 All Participants)

Item	Mean	SD	CV (%)
Reduce ICU mortality	2.38	0.83	34.9%
Increase ICU mortality	1.21	0.51	42.1%
Shorten ICU length of stay	2.70	0.53	19.6%
Increase ICU length of stay	1.2	0.50	41.7%
Increase ventilator-free days	2.63	0.66	25.1%
Decrease ventilator-free days	1.18	0.45	38.1%

Absolute CV differences across Delphi rounds

Outcome	Round-1 CV%	Round-2 CV%	Round-3 CV%	CV (R1-R2)	CV (R2-R3)
Protein Intake Dosage					
</= 0.8 g.kg.d	51.7%	45.4%	43%	6.30%	2.5%
0.8-1.2 g.kg.d	44.0%	39.5%	34.8%	4.5%	4.7%
1.2-1.5 g.kg.d	21.0%	27.3%	23.3%	-6.30%	4%
>/= 1.5 g.kg.d	44.8%	50.3%	45%	-5.50%	5.3%
Protein Intake Initiation					
< 24 hrs.	49.7%	46.5%	33%	3.20%	13.5%
24-48 hrs.	27.3%	29.8%	83%	-2.5%	53.2%
48-72 hrs.	46.9%	40.5%	75%	6.4%	34.5%
72 hrs.	56.9%	50.0%	76%	6.9%	26%
Patient-centred outcome					
Increase Muscle Mass	38.6%	38.6%	34.3%	0%	4.3%
Decrease Muscle Mass	30.4%				
Increase Muscle Function	27.1%	38.6%	30.5%	-11.5%	8.1%
Decrease Muscle Function	25.9%				

Mitigate Muscle Mass Losses	14.5%				
Mitigate Muscle Function Losses	19.6%	15.1%		4.6%	
Improve Physical Function Post-Discharge	17.4%	20.4%		-3%	
Worsen Physical Function Post-Discharge	34.7%				
Improve Quality of Life Post-Discharge	23.6%	24.2%	20%	-0.6%	4.2%
Worsen Quality of Life Post-Discharge	28.4%				
Reduce Risk of Discharge to Rehab	40.7%	40.1%	23.3%	0.6%	16.8%
Increase Risk of Discharge to Rehab	42.8%	34.7%	48.6%	8.1%	-13.9%
Conventional outcome					
Reduce ICU Mortality	23.2%	42.0%	29.6%	-18.80%	10.4%
Increase ICU Mortality	37.4%	28.7%	47.2%	8.7%	-18.5%
Shorten ICU LOS	27.9%	39.0%	41.1%	-11.1%	-11.1%
Lengthen ICU LOS	41.5%	37.4%	53.6%	4.1%	-16.2%
Increase Ventilator-Free Days	30.3%	39.0%	50.8%	-8.7%	-11.8%

Decrease Ventilator-Free Days	33.9%	37.7%	29.1%	-3.8%	8.6%
Decrease Sepsis Risk	34.6%	47.4%	41.4%	-12.8%	6%
Increase Sepsis Risk	28.7%	28.7%	44.9%	0%	-16.2%
Negatively Impact Renal Function	52.5%	45.4%	47.3%	7.1%	-1.9%
Positively Impact Renal Function	41.3%	47.4%	41.9%	-6.1%	5.5%
Enhance Wound Healing	10.9%				
Worsen Wound Healing	26.7%				
Effects of early protein supplementation in the IC					
Be harmful for septic patients	53.8%	51.9%	44.3%	1.90%	7.6%
Associated with less nutritional deficits	23.2%	29.4%	41.4%	-6.2%	-12%
Improve patient-centred functional outcomes	18.6%	31.2%	42.5%	-12.6%	-11.3%
Improve conventional clinical outcomes	24.8%	35.6%	44.8%	-10.8%	-9.2%

intermittent versus continuous protein provision in the ICU					
Intermittent, compared to continuous, provision could enhance the cellular process of muscle building	27.0%	29.7%	29.8%	-2.70%	-0.1%
Continuous, compared to intermittent, provision is more feasible in the ICU environment	31.4%	28.8%	24.7%	2.6%	4.1%
Intermittent, compared to continuous, enteral provision could have a negative effect on gastric function (e.g.,	36.0%	35.0%	31.8%	1%	3.2%

vomiting and aspirates)					
Exercise' (contractile activity) adjuvant to optimal protein intake could impact patient-centred functional outcomes					
Increase muscle mass	21.2%	31.6%	33.5%	-10.4%	-2.1%
Decrease muscle mass	33.0%				
Increase muscle function	7.5%				
Decrease muscle function	25.9%				
Mitigate muscle mass losses	15.2%				
Mitigate muscle function losses	19.3%	15.8%		3.5%	

Improve physical function following discharge	11.8%				
Worsen physical function following discharge	36.5%				
Improve quality of life following discharge	22.8%	17.2%		5.6%	
Worsen quality of life following discharge	31.8%				
Reduce risk of discharge to rehabilitation	31.8%	21.0%	18.5%	10.8%	2.5%
Increase risk of discharge to rehabilitation	37.5%	50.9%	46.2%	-13.4%	4.7%
Exercise' (contractile activity) adjuvant to optimal protein intake could impact					

conventional outcomes					
Reduce ICU mortality	26.6%	27%	34.9%	-0.4%	-7.9%
Increase ICU mortality	43.1%	35%	42.1%	8.1%	-7.1%
Shorten ICU length of stay	19.5%	23%	19.6%	-3.5%	3.4%
Increase ICU length of stay	43.2%	34%	41.7%	9.2%	-7.7%
Increase ventilator-free days	21.0%	35%	25.1%	-14%	9.9%
Decrease ventilator-free days	45.0%	47%	38.1%	-2%	8.9%

Red-highlighted values exceed the 20% threshold, indicating response inconsistency.
